# Competition between Cations via Classical Poisson–Nernst–Planck Models with Nonzero but Small Permanent Charges

**DOI:** 10.3390/membranes11040236

**Published:** 2021-03-26

**Authors:** Mingji Zhang

**Affiliations:** Department of Mathematics, New Mexico Institute of Mining and Technology, Socorro, NM 87801, USA; mingji.zhang@nmt.edu

**Keywords:** PNP, permanent charge, channel geometry, diffusion coefficient, individual fluxes, electroneutrality conditions, 34A26, 34B16, 34D15, 37D10, 92C35

## Abstract

We study a one-dimensional Poisson–Nernst–Planck system for ionic flow through a membrane channel. Nonzero but small permanent charge, the major structural quantity of an ion channel, is included in the model. Two cations with the same valences and one anion are included in the model, which provides more rich and complicated correlations/interactions between ions. The cross-section area of the channel is included in the system, and it provides certain information of the geometry of the three-dimensional channel, which is critical for our analysis. Geometric singular perturbation analysis is employed to establish the existence and local uniqueness of solutions to the system for small permanent charges. Treating the permanent charge as a small parameter, through regular perturbation analysis, we are able to derive approximations of the individual fluxes explicitly, and this allows us to study the competition between two cations, which is related to the selectivity phenomena of ion channels. Numerical simulations are performed to provide a more intuitive illustration of our analytical results, and they are consistent.

## 1. Introduction

Ion channels are large proteins embedded in cell membranes with a hole down their middle that provides a controllable path for electrodiffusion of ions (mainly Na${}^{+}$, K${}^{+}$, Ca${}^{++}$ and Cl${}^{-}$) through biological membranes, establishing communications among cells and the external environment [1,2,3]. In general, the study of ion channels consists of two related major topics: structures of ion channels and ionic flow properties.

The physical structure of ion channels is defined by the channel shape and the spacial distribution of permanent and polarization charge. Very often, the shape of a typical ion channel is approximated by a cylindrical-like domain with a non-uniform cross-section area. Within a large class of ion channels, amino acid side chains are distributed mainly over a relatively “short” and “narrow” portion of the channel, where acidic side chains contribute permanent negative charges and basic side chains contributes permanent positive charges, and this is analogous to the doping of semiconductor devices, e.g., bipolar PNP and NPN transistors [1,4].

With a given structure of an open channel, the main interest is to understand its electrodiffusion property. Mathematical analysis plays important and unique roles for generalizing and understanding the principles that allow control of electrodiffusion, explaining mechanics of observed biological phenomena and for discovering new ones, assuming a more or less explicit solution of the associated mathematical model can be obtained. However, in general, the latter is too much to expect. Recently, there have been some successes in mathematical analysis of Poisson–Nernst–Planck (PNP) models for ionic flows through membrane channels [5,6,7,8,9,10,11,12,13,14].

### 1.1. Poisson–Nernst–Planck Models for Ionic Flows

Considering the structural characteristics, the basic continuum model for ionic flows is the Poisson–Nernst–Planck system, which treats the aqueous medium as a dielectric continuum ([15,16,17,18] etc.). The PNP system can be derived as a reduced model from molecular dynamics [19], from Boltzmann equations [20], and from variational principles [21,22,23].

It is known that more sophisticated models [24,25,26,27,28,29,30,31] have also been developed which can model the physical problem more accurately, however, it is very challenging to examine their dynamics analytically and even computationally. Considering the key feature of the biological system, the PNP system represents an appropriate model for both analysis and numerical simulations of ionic flows.

The simplest PNP system is the classical Poisson–Nernst–Planck (cPNP) system that includes the ideal component $\mu_{k}^{id}\left(X\right)$ in (4) only. The ideal component $\mu_{k}^{id}$ contains contributions by considering ion particles as point charges and ignoring the ion-to-ion interaction. It has been shown by some numerical studies that classical PNP models provide good qualitative agreement with experimental data for I-V relations [20,32]. The classical PNP models have been simulated and analyzed extensively (see, e.g., [7,12,13,14,20,32,33,34,35,36,37,38,39]).

For ionic solutions with *n* ion species, the PNP system reads

(1) $$\begin{array}{c} \begin{array}{cc} \; & \nabla \cdot \left(\varepsilon_{r}\left(r\right)\varepsilon_{0}\nabla \Phi \right)=-e\left(\sum_{s=1}^{n}z_{s}C_{s}+Q\left(r\right)\right),\\ \; & \nabla \cdot J_{k}=0,\;-J_{k}=\frac{1}{k_{B}T}D_{k}\left(r\right)C_{k}\nabla \mu_{k},\;k=1,2,\cdots ,n, \end{array} \end{array}$$

where r∈Ω with Ω being a three-dimensional cylindrical-like domain representing the channel, Q(r) is the permanent charge density, $\varepsilon_{r}\left(r\right)$ is the relative dielectric coefficient, $\varepsilon_{0}$ is the vacuum permittivity, *e* is the elementary charge, $k_{B}$ is the Boltzmann constant, *T* is the absolute temperature; Φ is the electric potential. Furthermore, for the *k*th ion species, $C_{k}$ is the concentration, $z_{k}$ is the valence, $\mu_{k}$ is the electrochemical potential depending on Φ and $\left\{C_{j}\right\}$, $J_{k}$ is the flux density, and $D_{k}\left(r\right)$ is the diffusion coefficient.

Based on the fact that ion channels have narrow cross-sections relative to their lengths, reduction of the three-dimensional steady-state PNP systems (1) to quasi-one-dimensional models was first proposed in [40] and was rigorously justified in [36] for special cases. A quasi-one-dimensional steady-state PNP model takes the form

(2) $$\begin{array}{c} \begin{array}{cc} \; & \frac{1}{A(X)}\frac{d}{dX}\left(\varepsilon_{r}\left(X\right)\varepsilon_{0}A\left(X\right)\frac{d\Phi }{dX}\right)=-e\left(\sum_{s=1}^{n}z_{s}C_{s}+Q\left(X\right)\right),\\ \; & \frac{dJ_{k}}{dX}=0,\;-J_{k}=\frac{1}{k_{B}T}D_{k}\left(X\right)A\left(X\right)C_{k}\frac{d\mu_{k}}{dX},\;k=1,2,\cdots ,n, \end{array} \end{array}$$

where X∈[0,l] is the coordinate along the axis of the channel, A(X) is the area of cross-section of the channel over the location *X*.

Equipped with system (2), we impose the following boundary conditions (see, Ref. [7] for a reasoning), for k=1,2,⋯,n,

(3) $$\Phi \left(0\right)=V,\;\;C_{k}\left(0\right)=L_{k}>0;\;\Phi \left(l\right)=0,\;\;C_{k}\left(l\right)=R_{k}>0.$$

### 1.2. Electrochemical Potential

The electrochemical potential $\mu_{k}\left(X\right)$ for the *i*th ion species consists of the ideal component $\mu_{k}^{id}\left(X\right)$ and the excess component $\mu_{k}^{ex}\left(X\right)$: $\mu_{k}\left(X\right)=\mu_{k}^{id}\left(X\right)+\mu_{k}^{ex}\left(X\right)$, where

(4) $$\mu_{k}^{id}\left(X\right)=z_{k}e\Phi \left(X\right)+k_{B}T\ln\frac{C_{k}\left(X\right)}{C_{0}}$$

with some characteristic number density $C_{0}$ defined by

(5) $$\begin{array}{c} C_{0}=\max_{1\leq i\leq n}\left\{L_{i},R_{i},\underset{X\in [0,l]}{\sup}\left|Q\left(X\right)\right|\right\}. \end{array}$$

The cPNP system takes into consideration of the ideal component $\mu_{k}^{id}\left(x\right)$ only. This component reflects the collision between ion particles and the water molecules. It has been accepted that the cPNP system is a reasonable model in, for example, the dilute case under which the ion particles can be treated as point particles and the ion-to-ion interaction can be more or less ignored. The excess chemical potential $\mu_{k}^{ex}\left(x\right)$ accounts for the finite size effect of ions. We point out that, among many limitations, such as the “gating” phenomena, may not be captured by the simple cPNP model. However, the basic findings on dynamics of ionic flows and their dependence on the system parameters, particularly, the permanent charges, the channel geometry, the ratios of boundary concentrations, and the ratios of diffusion constants, provide important insights into the mechanism of ion channels and better understandings of ionic flow properties. More importantly, some of them are non-intuitive, and deserve further studies. More structural detail and more correlations between ions should be taken into considerations in PNP models such as those including various potentials for ion-to-ion interaction accounting for ion size effects ([5,21,41,42,43,44] etc.).

### 1.3. Permanent Charge

The spatial distribution of side chains in a specific channel defines the permanent charge of the channel. While some information may be obtained by ignoring the permanent charge and focusing on the effects of boundary conditions, the valences and sizes of ions, etc., we believe that different channel types differ mainly in the distribution of permanent charge [3]. To better understand the importance of the relation of ionic flows and permanent charges, we remark that the role of permanent charges in membrane channels is similar to the role of doping profiles in semiconductor devices. Semiconductor devices are similar to membrane channels in the way that they both use atomic-scale structures to control macroscopic flows from one reservoir to another. Ions move a lot like quasi-particles move in semiconductors. Roughly, holes and electrons are the cations and anions of semiconductors. Semiconductor technology depends on the control of migration and diffusion of quasi-particles of charge in transistors and integrated circuits. Doping is the process of adding impurities into intrinsic semiconductors to modulate its electrical, optical, and structural properties [45,46]. One may roughly understand in the following sense, doping provides the charges that acid and basic side chains provide in a protein channel. For both ion channels and semiconductors, permanent charges add an additional component—probably the most important one—to their rich behavior.

In general, the permanent charge Q(X) is modeled by a piecewise constant function, that is, we assume, for a partition $X_{0}=0<X_{1}<\cdots <X_{m-1}<X_{m}=l$ of [0,l] into *m* subintervals, $Q\left(X\right)=Q_{j}$ for $x\in (X_{j-1},X_{j})$ where $Q_{j}$’s are constants with $Q_{1}=Q_{m}=0$ (the intervals $\left[X_{0},X_{1}\right]$ and $\left[X_{m-1},X_{m}\right]$ are viewed as the reservoirs where there is no permanent charge). In this work, we take the following model for Q(x)

(6) $$Q\left(X\right)=\left\{\begin{array}{cc} 0, & X_{0}<X<X_{1},\\ Q_{0}, & X_{1}<X<X_{2},\\ 0, & X_{2}<X<X_{3}, \end{array}\right.$$

where $X_{0}=0,\;X_{3}=l$ and $Q_{0}$ is some nonzero constant.

### 1.4. Comparison with Some Existing Works

The current work follows a similar dynamical system framework as that employed in [6,7,9,10,11] to establish the existence and uniqueness result of the problem. However, compared to these works, our set-ups are much more challenging and more realistic, more importantly, the specific structure of our model allows us to obtain detailed description of the nonlinear interplay among different system parameters. This is far beyond the existence and uniqueness result. To be specific, our model includes three ion species, two positively charged with the same valences, and one negatively charged (in [7,9,10], only two oppositely charged particles are included, selectivity of cations, one of the most relevant biological properties of ion channels cannot be described); and a profile of nonzero but small permanent charges (in [6], it includes three ion species but with zero permanent charges, the effects on ionic flows from the two key structures of ion channels: channel geometry and distribution of permanent charges, cannot be examined, while this could provide crucial insights for the selectivity of cations through membrane channels). In [11], the author extended the work in [7] and established the existence and local uniqueness of the classical PNP system with *n* ion species.

The work, in some sense, is motivated by [9], and there are some similarities in the treatment. More precisely, both of the works employ regular perturbation analysis to derive the explicit expressions of the individual fluxes up to the first order in the small permanent charge, which is reflected in Section 2.2 in the current work. However, the derivation and the following analysis is much more challenging due to the nonlinearity of the individual fluxes in the potential *V* (in [9], the individual fluxes are linear in the potential *V*). The nonlinearity of the individual fluxes in the potential provides much more rich dynamics of ionic flows, and demonstates more complicated nonlinear interaction among the system parameters, which is addressed in Section 3.1. Meanwhile, this indicates that our work provides a better understanding of the mechanism of ionic flows through single ion channels, which is necessary and important for future studies of ion channel problems.

### 1.5. Main Results

For convenience, we briefly summarize our main results as follows with j,k=1,2,3.

(i) Constructing a singular orbit of the limiting PNP system (ε→0) over the whole interval [0,1], which is a union of singular orbits over the subintervals [0,a],[a,b] and [b,1]. Over each subinterval, the singular orbit consists of two boundary/internal layers and a regular layer; see Proposition 2 and Lemma 2 in Section 2.1.1 for the singular over [0,a], in Proposition 4 and Lemma 3 for the one over [a,b] in Section 2.1.2, and Proposition 5 and Lemma 4 for the one over [b,1] in Section 2.1.3.
(ii) Establishing the existence and local uniqueness result of the underlying PNP system (ε>0 but small); see Theorem 1 in Section 2.1.5.
(iii) Obtaining the zeroth order and first order (in $Q_{0}$) solutions of system (40) and (41), crucial to derive explicit expressions of the individual fluxes up to the first order in $Q_{0}$; see Propositions 6 and 7 in Section 2.2.
(iv) The sign of *A* and 1−B, critical for our analysis in Section 3.1; see Lemmas 7 and 8 in Section 2.2.
(v) Study on competition between cations in terms of $J_{1,2}^{1}=D_{1}J_{11}-D_{2}J_{21}$ from two directions: the sign of $J_{1,2}^{1}$ and the monotonicity of $J_{1,2}^{1}$ in the electric potential *V*, based on distinct interplays among $\frac{D_{1}}{D_{2}},\;\frac{L_{2}}{L_{1}}$ and $\frac{R_{2}}{R_{1}}$ consisting of three cases
  (v1) Case study with $\frac{D_{1}}{D_{2}}=\frac{L_{2}}{L_{1}}$; see Theorems 2–4 in Section 3.1.1.
  (v2) Case study with $\frac{D_{1}}{D_{2}}<\min\left\{\frac{L_{2}}{L_{1}},\frac{R_{2}}{R_{1}}\right\}$; see Theorems 5–7 in Section 3.1.2.
  (v3) Case study with $\frac{R_{2}}{R_{1}}<\frac{D_{1}}{D_{2}}<\frac{L_{2}}{L_{1}}$; see Theorems 8 and 9 in Section 3.1.3.
(vi) Analysis on the magnitude of $J_{1,2}$, equivalent to examine the sign of $J_{1,2}^{0}J_{1,2}^{1}$, where $J_{1,2}^{0}=D_{1}J_{10}-D_{2}J_{20}$; see Theorems 10 and 11 in Section 3.1.4.

**Remark** **1.** *In (v), there are actually another three cases: (1) $\frac{D_{1}}{D_{2}}=\frac{R_{2}}{R_{1}}$; (2) $\frac{D_{1}}{D_{2}}>\max\left\{\frac{L_{2}}{L_{1}},\frac{R_{2}}{R_{1}}\right\}$; and (3) $\frac{L_{2}}{L_{1}}<\frac{D_{1}}{D_{2}}<\frac{R_{2}}{R_{1}}$. The results and arguments are similar to those corresponding to the case stated in (vi-1)–(vi-3), and are not included in this work. Interested readers can study them following our discussions detailed in Section 3.1.*

### 1.6. Problem Set-Up

For definiteness, we will take the following setting in this work:

(A1). We consider three ion species (n=3) with $z_{1}=z_{2}=z>0$ and $z_{3}<0$.
(A2). The permanent charge is defined as in (6).
(A3). For the electrochemical potential $\mu_{i}$, we only include the ideal component $\mu_{i}^{id}$ as in (4).
(A4). We assume $\varepsilon_{r}\left(X\right)=\varepsilon_{r}$ and $D_{i}\left(X\right)=D_{i}$.

In the sequel, we will assume (A1)–(A4). We first make a dimensionless rescaling following [3]. With $C_{0}$ given in (5), let

(7) $$\begin{array}{c} \begin{array}{cc} \; & \varepsilon^{2}=\frac{\varepsilon_{r}\varepsilon_{0}k_{B}T}{e^{2}l^{2}C_{0}},\;x=\frac{X}{l},\;h\left(x\right)=\frac{A(X)}{l^{2}},\;D_{i}=lC_{0}D_{i},\;\phi \left(x\right)=\frac{e}{k_{B}T}\Phi \left(X\right),\\ \; & c_{i}\left(x\right)=\frac{C_{i}\left(X\right)}{C_{0}},\;J_{i}=\frac{J_{i}}{D_{i}},\;V=\frac{e}{k_{B}T}V,\;L_{i}=\frac{L_{i}}{C_{0}},R_{i}=\frac{R_{i}}{C_{0}}. \end{array} \end{array}$$

The BVP (2) and (3) then becomes (noting that $z_{1}=z_{2}=z$)

(8) $$\begin{array}{c} \begin{array}{cc} \; & \frac{\varepsilon^{2}}{h(x)}\frac{d}{dx}\left(h\left(x\right)\frac{d}{dx}\phi \right)=-zc_{1}-zc_{2}-z_{3}c_{3}-Q\left(x\right),\;\frac{dc_{1}}{dx}+zc_{1}\frac{d\phi }{dx}=-\frac{J_{1}}{h(x)},\\ \; & \frac{dc_{2}}{dx}+zc_{2}\frac{d\phi }{dx}=-\frac{J_{2}}{h(x)},\;\frac{dc_{3}}{dx}+z_{3}c_{3}\frac{d\phi }{dx}=-\frac{J_{3}}{h(x)},\;\frac{dJ_{k}}{dx}=0, \end{array} \end{array}$$

with the boundary conditions, for i=1,2,3,

(9) $$\phi \left(0\right)=V,\;\;c_{i}\left(0\right)=L_{i}>0;\;\;\phi \left(1\right)=0,\;\;c_{i}\left(1\right)=R_{i}>0.$$

We comment that the dimensionless parameter ε defined in (1.6) as $\varepsilon =\frac{1}{l}\sqrt{\frac{\varepsilon_{r}\varepsilon_{0}k_{B}T}{e^{2}C_{0}}}$ is directly related to the ratio $\kappa_{D}/l,$ where $\kappa_{D}=\sqrt{\frac{\varepsilon_{r}\varepsilon_{0}k_{B}T}{\sum_{j}{\left(z_{j}e\right)}^{2}C_{j}}}$ is the Debye length; in particular, $\varepsilon =\kappa_{D}/l$ when $z_{j}^{2}=1$ and $C_{j}=C_{0}$. Typically, the parameter ε is small due to the fact that the two variables *l*, the length of the channel, and $C_{0}$, some characteristic number density could be very large. For many cases, the value of ε is of order $O(10^{-3})$ (see [47] for a more detailed description).

## 2. Methods

### 2.1. Geometric Singular Perturbation Theory for (8) and (9)

We first rewrite system (8) into a standard form for singularly perturbed systems and convert the boundary value problem (8) and (9) to a connection problem.

Upon introducing $u=\varepsilon \dot{\phi }$ and τ=x. System (8) becomes

(10) $$\begin{array}{c} \begin{array}{cc} \varepsilon \dot{\phi }= & u,\;\varepsilon \dot{u}=-zc_{1}-zc_{2}-z_{3}c_{3}-Q\left(x\right)-\varepsilon \frac{h_{\tau }\left(\tau \right)}{h(\tau )}u,\;\varepsilon {\dot{c}}_{1}=-zc_{1}u-\frac{\varepsilon }{h(\tau )}J_{1},\\ \varepsilon {\dot{c}}_{2}= & -zc_{2}u-\frac{\varepsilon }{h(\tau )}J_{2},\;\varepsilon {\dot{c}}_{3}=-z_{3}c_{3}u-\frac{\varepsilon }{h(\tau )}J_{3},\;{\dot{J}}_{1}={\dot{J}}_{2}={\dot{J}}_{3}=0,\;\dot{\tau }=1, \end{array} \end{array}$$

where overdot denotes the derivative with respect to the variable *x*.

System (10) will be treated as a singularly perturbed system with ε as the singular parameter. Its phase space is $R^{9}$ with state variables $\left(\phi ,u,c_{1},c_{2},c_{3},J_{1},J_{2},J_{3},\tau \right)$.

For ε>0, the rescaling x=εξ of the independent variable *x* gives rise to

(11) $$\begin{array}{c} \begin{array}{cc} \phi^{\prime }= & u,\;u^{\prime }=-zc_{1}-zc_{2}-z_{3}c_{3}-Q\left(x\right)-\varepsilon \frac{h_{\tau }\left(\tau \right)}{h(\tau )}u,\;c_{1}^{\prime }=-zc_{1}u-\frac{\varepsilon }{h(\tau )}J_{1},\\ c_{2}^{\prime }= & -zc_{2}u-\frac{\varepsilon }{h(\tau )}J_{2},\;c_{3}^{\prime }=-z_{3}c_{3}u-\frac{\varepsilon }{h(\tau )}J_{3},\;J_{1}^{\prime }=J_{2}^{\prime }=J_{3}^{\prime }=0,\;\tau^{\prime }=\varepsilon , \end{array} \end{array}$$

where prime denotes the derivative with respect to the variable ξ.

We comment that for ε>0, systems (10) and (11) have exactly the same phase portrait. However, their limiting systems at ε=0 are different. The limiting system of (10) is called the limiting slow system, whose orbits are called slow orbits or regular layers. The limiting system of (11) is the limiting fast system, whose orbits are called fast orbits or singular (boundary and/or internal) layers. By a singular orbit of system (10) or (11), we mean a continuous and piecewise smooth curve in $R^{9}$ that is a union of finitely many slow and fast orbits. Very often, limiting slow and fast systems provide complementary information on state variables. Correspondingly, the main task of singularly perturbed problems is to patch the limiting information together to form a solution for the entire ε>0 system.

Let $B_{L}$ and $B_{R}$ be the subsets of the phase space $R^{9}$ defined by

(12) $$\begin{array}{c} \begin{array}{cc} B_{L}= & \{\left(V,u,L_{1},L_{2},L_{3},J_{1},J_{2},J_{3},0\right)\in R^{9}:\mathrm{arbitrary}\;u,J_{1},J_{2},J_{3}\},\\ B_{R}= & \{\left(0,u,R_{1},R_{2},R_{3},J_{1},J_{2},J_{3},1\right)\in R^{9}:\mathrm{arbitrary}\;u,J_{1},J_{2},J_{3}\}. \end{array} \end{array}$$

Then the original boundary value problem is equivalent to a connection problem, namely, finding a solution of (10) or (11) from $B_{L}$ to $B_{R}$ (see, for example, [48]).

Due to the jumps of the permanent charge function (6) at x=a and x=b, we split the interval [0,1] into three subintervals [0,a],[a,b] and [b,1], where the intervals [0,a] and [b,1] represent the reservoirs, and the interval [a,b] represents the channel. To construct a singular orbit over the whole interval [0,1], we first construct a singular orbit on each of the subintervals. To get started, we preassign the values of $\phi ,\;c_{1},\;c_{2}$ and $c_{3}$ at x=a and x=b as follows:

(13) $$\begin{array}{c} \begin{array}{cc} \; & \phi \left(a\right)=\phi^{\left[a\right]},\;c_{k}\left(a\right)=c_{k}^{\left[a\right]};\;\phi \left(b\right)=\phi^{\left[b\right]},\;c_{k}\left(b\right)=c_{k}^{\left[b\right]},\;k=1,2,3. \end{array} \end{array}$$

These eight unknown values will be determined along our construction of a singular orbit on the whole interval [0,1].

(i) The singular orbit on [0,a] consists of two boundary layers $\Gamma_{l}^{0}$ and $\Gamma_{l}^{a}$ and one regular layer $\Lambda_{l}$ with $\left(\phi ,c_{1},c_{2},c_{3},\tau \right)$ being
  $$\left(V,L_{1},L_{2},L_{3},0\right)\;\mathrm{at}\;x=0\;\mathrm{and}\;\left(\phi^{\left[a\right]},c_{1}^{\left[a\right]},c_{2}^{\left[a\right]},c_{3}^{\left[a\right]},a\right)\;\mathrm{at}\;x=a.$$
  In particular, given $(\phi^{\left[a\right]},c_{1}^{\left[a\right]},c_{2}^{\left[a\right]},c_{3}^{\left[a\right]}),$ the flux densities $J_{k}^{l}$ and the value $u_{l}\left(a\right)$ are uniquely determined (see Section 2.1.1).
(ii) The singular orbit on [a,b] consists of two boundary layers $\Gamma_{m}^{a}$ and $\Gamma_{m}^{b}$ and one regular layer $\Lambda_{m}$ with $\left(\phi ,c_{1},c_{2},c_{3},\tau \right)$ being
  $$\left(\phi^{\left[a\right]},c_{1}^{\left[a\right]},c_{2}^{\left[a\right]},c_{3}^{\left[a\right]},a\right)\;\mathrm{at}\;x=a\;\mathrm{and}\;\left(\phi^{\left[b\right]},c_{1}^{\left[b\right]},c_{2}^{\left[b\right]},c_{3}^{\left[b\right]},b\right)\;\mathrm{at}\;x=b.$$
  In particular, given $\left(\phi^{\left[a\right]},c_{1}^{\left[a\right]},c_{2}^{\left[a\right]},c_{3}^{\left[a\right]}\right)$ and $\left(\phi^{\left[b\right]},c_{1}^{\left[b\right]},c_{2}^{\left[b\right]},c_{3}^{\left[b\right]}\right)$, the flux densities $J_{k}^{m}$ and the value $u_{m}\left(a\right)$ and $u_{m}\left(b\right)$ are uniquely determined (see Section 2.1.2).
(iii) The singular orbit on [b,1] consists of two boundary layers $\Gamma_{r}^{b}$ and $\Gamma_{r}^{1}$ and one regular layer $\Lambda_{r}$ with $\left(\phi ,c_{1},c_{2},c_{3},\tau \right)$ being
  $$\left(\phi^{\left[b\right]},c_{1}^{\left[b\right]},c_{2}^{\left[b\right]},c_{3}^{\left[b\right]},b\right)\;\mathrm{at}\;x=b\;\mathrm{and}\;\left(0,R_{1},R_{2},R_{3},1\right)\;\mathrm{at}\;x=1.$$
  In particular, given $\left(\phi^{\left[b\right]},c_{1}^{\left[b\right]},c_{2}^{\left[b\right]},c_{3}^{\left[b\right]}\right)$, the flux densities $J_{k}^{r}$ and the value $u_{r}\left(b\right)$ are uniquely determined (see Section 2.1.3).

To obtain a singular orbit on the whole interval [0,1], one need

(14) $$\begin{array}{c} \begin{array}{cc} \; & J_{k}^{l}=J_{k}^{m}=J_{k}^{r},\;u_{l}\left(a\right)=u_{m}\left(a\right),\;u_{m}\left(b\right)=u_{r}\left(b\right),\;k=1,2,3. \end{array} \end{array}$$

This consists of eight conditions. The number of conditions is exactly the same as the number of unknowns in (13).

The singular orbit constructed for problem (10) associated to $B_{L}$ and $B_{R}$ consists of nine pieces $\Gamma_{l}^{0}\cup \Lambda_{l}\cup \Gamma_{l}^{a}\cup \Gamma_{m}^{a}\cup \Lambda_{m}\cup \Gamma_{m}^{b}\cup \Gamma_{r}^{b}\cup \Lambda_{r}\cup \Gamma_{r}^{1}$. Once a singular orbit is constructed, one then can apply the geometric singular perturbation theory, such as Exchange Lemma, to show that, for ε>0 but small, there is a unique solution that is close to the singular orbit.

In this work, we will examine the competition between cations due to the nonlinear interaction among channel geometry, small permanent charges, diffusion coefficients and boundary conditions, which can be extracted from the matching conditions (14) (for simplicity, we still use $\left(\phi ,u,c_{1},c_{2},c_{3},J_{1},J_{2},J_{3}\right)$ for the zeroth order system in ε).

We remark that $u_{l}\left(a\right)$, $u_{m}\left(a\right)$, $u_{m}\left(b\right)$, $u_{r}\left(b\right)$, $J_{k}^{l}$, $J_{k}^{m}$, $J_{k}^{r}$ are actually the functions of the unknowns $\phi^{\left[a\right]},\;c_{k}^{\left[a\right]},\;\phi^{\left[b\right]},\;c_{k}^{\left[b\right]}$ with the parameter $Q_{0}$. Furthermore, for simplicity, in the following analysis, we will use $J_{k}$ to denote $J_{k}^{l},\;J_{k}^{m}$ and $J_{k}^{r}$, respectively.

Once a solution for $\left(\phi^{\left[a\right]},c_{1}^{\left[a\right]},c_{2}^{\left[a\right]},c_{3}^{\left[a\right]};\phi^{\left[b\right]},c_{1}^{\left[b\right]},c_{2}^{\left[b\right]},c_{3}^{\left[b\right]}\right)$ is determined, one then can derive the zeroth order (in ε) individual fluxes $J_{k}\left(Q_{0}\right)=D_{k}J_{k}\left(Q_{0}\right)$. Through our following discussions, we always assume the so-called electroneutrality boundary concentration conditions

(15) $$\begin{array}{c} z\left(L_{1}+L_{2}\right)+z_{3}L_{3}=0,\;\mathrm{and}\;z\left(R_{1}+R_{2}\right)+z_{3}R_{3}=0. \end{array}$$

For simplicity, we also introduce

(16) $$\begin{array}{c} \begin{array}{cc} \; & L=L_{1}+L_{2},\;R=R_{1}+R_{2},\;L_{d}=D_{1}L_{1}-D_{2}L_{2},\;R_{d}=D_{1}R_{1}-D_{2}R_{2},\\ \; & C^{\left[a\right]}=c_{1}^{\left[a\right]}+c_{2}^{\left[a\right]},\;C^{\left[b\right]}=c_{1}^{\left[b\right]}+c_{2}^{\left[b\right]},\;C^{\left[a,l\right]}=c_{1}^{\left[a,l\right]}+c_{2}^{\left[a,l\right]},\\ \; & C^{\left[a,m\right]}=c_{1}^{\left[a,m\right]}+c_{2}^{\left[a,m\right]},\;C^{\left[b,m\right]}=c_{1}^{\left[b,m\right]}+c_{2}^{\left[b,m\right]},\;C^{\left[b,r\right]}=c_{1}^{\left[b,r\right]}+c_{2}^{\left[b,r\right]}. \end{array} \end{array}$$

#### 2.1.1. Singular Orbit on [0,a] with Q(x)=0

Following (13), we introduce

$$B_{a}=\left\{\left(\phi^{\left[a\right]},u,c_{1}^{\left[a\right]},c_{2}^{\left[a\right]},c_{3}^{\left[a\right]},J_{1},J_{2},J_{3},a\right)\in R^{9}:u,J_{1},J_{2},J_{3},\;\mathrm{arbitrary}\right\}.$$

We now construct a singular orbit on [0,a] that connects $B_{L}$ to $B_{a},$ which generally consist of two boundary layers and a regular layer (see [7,10,11,43]). Over the subinterval [0,a], the permanent charge is zero because we review [0,a] as one of the reservoirs. However, the nonzero $Q_{0}$ over the subinterval [a,b] will affect the solution on [0,a] and [b,1] (another reservoir with zero permanent charge) through the matching conditions imposed on $\phi^{\left[a\right]},c_{1}^{\left[a\right]},c_{2}^{\left[a\right]},c_{3}^{\left[a\right]}$ and $\phi^{\left[b\right]},c_{1}^{\left[b\right]},c_{2}^{\left[b\right]},c_{3}^{\left[b\right]}$ to construct the singular orbit over the whole interval [0,1].

**Limiting fast dynamics and boundary layers on [0,a]** Setting ε=0 in (10), we get the so-called slow manifold,

$$\begin{array}{c} Z_{l}=\left\{u=0,zc_{1}+zc_{2}+z_{3}c_{3}=0\right\}. \end{array}$$

Setting ε=0 in (11), we get the limiting fast system,

(17) $$\begin{array}{c} \begin{array}{cc} \phi^{\prime }= & u,\;u^{\prime }=-zc_{1}-zc_{2}-z_{3}c_{3},\;c_{1}^{\prime }=-zc_{1}u,\\ c_{2}^{\prime }= & -zc_{2}u,\;c_{3}^{\prime }=-z_{3}c_{3}u,\;J_{1}^{\prime }=J_{2}^{\prime }=J_{3}^{\prime }=0,\;\tau^{\prime }=0. \end{array} \end{array}$$

Note that the slow manifold $Z_{l}$ is the set of equilibria of (17). The following can be established directly [11].

**Lemma** **1.** *For system (17), the slow manifold $Z_{l}$ is normally hyperbolic.*

**Proof.**  The slow manifold $Z_{l}$ is precisely the set of equilibria of (17). The linearization of (17) at each point of $\left(\phi ,0,c_{1},c_{2},c_{3},J_{1},J_{2},J_{3},\tau \right)\in Z_{l}$ has seven zero eigenvalues whose generalized eigenspace is the tangent space of the seven-dimensional slow manifold $Z_{l}$ of equilibria, and the other two eigenvalues are $\pm \sqrt{z_{1}^{2}c_{1}+z_{2}^{2}c_{2}+z_{3}^{2}c_{3}}$, whose eigenvectors are not tangent to $Z_{l}$ (Recall that $c_{i}$’s are concentrations and we are only interested in positive ones). Thus, $Z_{l}$ is normally hyperbolic. □

The theory of normally hyperbolic invariant manifolds [49] states that there exists eight-dimensional stable manifold $W^{s}\left(Z_{l}\right)$ of $Z_{l}$ that consists of points approaching $Z_{l}$ in forward time; and there exists eight-dimensional unstable manifold $W^{u}\left(Z_{l}\right)$ of $Z_{l}$ that consists of points approaching $Z_{l}$ in backward time. Let $M_{l}^{0}$ be the collection of orbits from $B_{L}$ in forward time under the flow of system (17) and $M_{l}^{a}$ be the collection of orbits from $B_{a}$ in backward time under the flow of system (17). Then, for a singular orbit connecting $B_{L}$ to $B_{a}$, the boundary layer $\Gamma_{l}^{0}$ at x=0 must lie in $N_{l}^{0}=M_{l}^{0}\cap W^{s}\left(Z_{l}\right)$ and the boundary layer $\Gamma_{l}^{a}$ at x=a must lie in $N_{l}^{a}=M_{l}^{a}\cap W^{u}\left(Z_{l}\right)$. In this subsection, we will determine the boundary layers $N_{l}^{0}$ and $N_{l}^{a}$, and their landing points $\omega (N_{l}^{0})$ and $\alpha (N_{l}^{a})$ on the slow manifold $Z_{l}$. The regular layer, determined by the limiting slow system, will lie in $Z_{l}$ and connect the landing points $\omega (N_{l}^{0})$ at x=0 and $\alpha (N_{l}^{a})$ at x=a.

First, one has

**Proposition** **1.** *The following functions are the first integrals of system (17),*

$$\begin{array}{cc} H_{1}= & \ln c_{1}+z\phi ,\;H_{2}=\ln c_{2}+z\phi ,\;H_{3}=\ln c_{3}+z_{3}\phi ,\;H_{4}=\frac{u^{2}}{2}-c_{1}-c_{2}-c_{3},\\ H_{5}= & J_{1},\;H_{6}=J_{2},\;H_{7}=J_{3},\;H_{8}=\tau . \end{array}$$

**Proof.**  This can be verified directly. □

For the landing points $\omega (N_{l}^{0})$ and $\alpha (N_{l}^{a})$, following the similar outline as those in [7,11], one has

**Proposition** **2.** *Assume the condition (15), one has*

(i) *The stable manifold $W^{s}\left(Z_{l}\right)$ intersects $B_{L}$ transversally at points $\left(V,u_{r}^{0},L_{1},L_{2},L_{3},J_{1},J_{2},J_{3},0\right),$ and the ω-limit set of $N_{l}^{0}=M_{l}^{0}⋂W^{s}\left(Z_{l}\right)$ is*
  $$\omega \left(N_{l}^{0}\right)=\left\{(\phi^{L},0,c_{1}^{L},c_{2}^{L},c_{3}^{L},J_{1},J_{2},J_{3},0)\right\},$$
  *where $J_{i}$ for i=1,2,3 are arbitrary, and*
  $$\begin{array}{cc} \phi^{L}= & V,\;c_{1}^{L}=L_{1},\;c_{2}^{L}=L_{2},\;c_{3}^{L}=L_{3},\;u_{r}^{0}=0. \end{array}$$
(ii) *The unstable manifold $W^{u}\left(Z_{l}\right)$ intersects $B_{a}$ transversally at points $\left(\phi^{\left[a\right]},u_{l}\left(a\right),c_{1}^{\left[a\right]},c_{2}^{\left[a\right]},c_{3}^{\left[a\right]},J_{1},J_{2},J_{3},a\right),$ and the α-limit set of $N_{l}^{a}=M_{l}^{a}⋂W^{u}\left(Z_{l}\right)$ is*
  $$\alpha \left(N_{l}^{a}\right)=\left\{(\phi^{\left[a,l\right]},0,c_{1}^{\left[a,l\right]},c_{2}^{\left[a,l\right]},c_{3}^{\left[a,l\right]},J_{1},J_{2},J_{3},a)\right\},$$
  *where $J_{i}$ for i=1,2,3 are arbitrary, and for k=1,2,*
  $$\begin{array}{cc} \phi^{\left[a,l\right]}= & \phi^{a}-\frac{1}{z-z_{3}}\ln\frac{-z_{3}c_{3}^{\left[a\right]}}{zC^{\left[a\right]}},\;c_{k}^{\left[a,l\right]}=c_{k}^{\left[a\right]}{\left(\frac{-z_{3}c_{3}^{\left[a\right]}}{zC^{\left[a\right]}}\right)}^{\frac{z}{z-z_{3}}},\;c_{3}^{\left[a,l\right]}=c_{3}^{\left[a\right]}{\left(\frac{-z_{3}c_{3}^{\left[a\right]}}{zC^{\left[a\right]}}\right)}^{\frac{z_{3}}{z-z_{3}}} \end{array}$$
  *and*
  (18) $$\begin{array}{c} \begin{array}{cc} \; & u_{l}\left(a\right)=sgn\left(\phi^{\left[a\right]}-\phi^{\left[a,l\right]}\right)\sqrt{2C^{\left[a\right]}\left(1-e^{z(\phi^{\left[a\right]}-\phi^{\left[a,l\right]})}\right)+2c_{3}^{a}\left(1-e^{z_{3}\left(\phi^{\left[a\right]}-\phi^{\left[a,l\right]}\right)}\right)}. \end{array} \end{array}$$
(iii) *The boundary layer $\Gamma_{l}^{0}$ at x=0 is determined up to $\left(J_{1},J_{2},J_{3}\right)$ as follows: the ϕ-component satisfies the Hamiltonian system*
  $$\phi^{\prime \prime }+zL_{1}e^{z(V-\phi )}+zL_{2}e^{z(V-\phi )}+z_{3}L_{3}e^{z_{3}\left(V-\phi \right)}=0,$$
  *together with ϕ(0)=V and $\phi \left(\xi \right)\to \phi^{L}$ as $\xi \to \infty ,\;u\left(\xi \right)=\phi^{\prime }\left(\xi \right),$ and*
  $$c_{1}\left(\xi \right)=L_{1}e^{z(V-\phi (\xi ))},\;c_{2}\left(\xi \right)=L_{2}e^{z(V-\phi (\xi ))},\;c_{3}\left(\xi \right)=L_{3}e^{z_{3}\left(V-\phi \left(\xi \right)\right)}.$$
*Similarly, the boundary layer $\Gamma_{l}^{a}$ at x=a is determined in the following way: the ϕ-component satisfies the Hamiltonian system*
  $$\phi^{\prime \prime }+zc_{1}^{\left[a\right]}e^{z(\phi^{\left[a\right]}-\phi )}+zc_{2}^{\left[a\right]}e^{z(\phi^{\left[a\right]}-\phi )}+z_{3}c_{3}^{\left[a\right]}e^{z_{3}\left(\phi^{\left[a\right]}-\phi \right)}=0,$$
  *together with $\phi \left(a\right)=\phi^{\left[a\right]}$ and $\phi \left(\xi \right)\to \phi^{\left[a,l\right]}$ as $\xi \to -\infty ,\;u\left(\xi \right)=\phi^{\prime }\left(\xi \right),$ and*
  $$c_{1}\left(\xi \right)=c_{1}^{\left[a\right]}e^{z(\phi^{\left[a\right]}-\phi \left(\xi \right))},\;c_{2}\left(\xi \right)=c_{2}^{\left[a\right]}e^{z(\phi^{\left[a\right]}-\phi \left(\xi \right))},\;c_{3}\left(\xi \right)=c_{3}^{\left[a\right]}e^{z_{3}\left(\phi^{\left[a\right]}-\phi \left(\xi \right)\right)}.$$

**Limiting slow dynamics and regular layers on [0,a]** For convenience, we introduce $T^{m},\;T^{c}$ and H(x) defined as

(19) $$T^{m}=J_{1}+J_{2}+J_{3},\;T^{c}=z\left(J_{1}+J_{2}\right)+z_{3}J_{3},\;H\left(x\right)=\int_{0}^{x}h^{-1}\left(\tau \right)d\tau .$$

Next we construct the regular layer on $Z_{l}$ that connects $\omega (N_{r}^{0})$ and $\alpha (N_{l}^{a})$. Note that, for ε=0, system (10) loses most information. To remedy this degeneracy [7,10,11], we make a rescaling u=εp and $-zc_{1}-zc_{2}-z_{3}c_{3}=\varepsilon q$ in system (10). In term of the new variables, system (10) becomes

$$\begin{array}{c} \begin{array}{cc} \dot{\phi }= & p,\;\varepsilon \dot{p}=q-\varepsilon \frac{h^{\prime }\left(\tau \right)}{h(\tau )}p,\;\varepsilon \dot{q}=\left(z\left(z-z_{3}\right)\left(c_{1}+c_{2}\right)-\varepsilon z_{3}q\right)p+\frac{T^{c}}{h(\tau )},\\ {\dot{c}}_{1}= & -zc_{1}p-\frac{J_{1}}{h(\tau )},\;{\dot{c}}_{2}=-zc_{2}p-\frac{J_{2}}{h(\tau )},\;{\dot{J}}_{k}=0,\;\dot{\tau }=1. \end{array} \end{array}$$

It is again a singular perturbation problem and its limiting slow system is

(20) $$\begin{array}{c} \begin{array}{cc} \dot{\phi }= & p,\;q=0,\;p=-\frac{T^{c}}{z\left(z-z_{3}\right)\left(c_{1}+c_{2}\right)h\left(\tau \right)},\\ {\dot{c}}_{1}= & -zc_{1}p-\frac{J_{1}}{h(\tau )},\;{\dot{c}}_{2}=-zc_{2}p-\frac{J_{2}}{h(\tau )},\;{\dot{J}}_{k}=0,\;\dot{\tau }=1. \end{array} \end{array}$$

For system (20), the slow manifold is $S_{l}=\left\{q=0,\;p=-\frac{T^{c}}{z\left(z-z_{3}\right)\left(c_{1}+c_{2}\right)h\left(\tau \right)}\right\}.$ Therefore, the limiting slow system on $S_{l}$ is given by

(21) $$\begin{array}{c} \begin{array}{cc} \dot{\phi }= & -\frac{T^{c}}{z\left(z-z_{3}\right)\left(c_{1}+c_{2}\right)h\left(\tau \right)},\;{\dot{c}}_{1}=\frac{T^{c}}{\left(z-z_{3}\right)\left(c_{1}+c_{2}\right)h\left(\tau \right)}c_{1}-\frac{J_{1}}{h(\tau )},\\ {\dot{c}}_{2}= & \frac{T^{c}}{\left(z-z_{3}\right)\left(c_{1}+c_{2}\right)h\left(\tau \right)}c_{2}-\frac{J_{2}}{h(\tau )},\;{\dot{J}}_{k}=0,\;\dot{\tau }=1. \end{array} \end{array}$$

For system (21), one has

**Lemma** **2.** *There is a unique solution $\left(\phi \left(x\right),c_{1}\left(x\right),c_{2}\left(x\right),J_{1},J_{2},J_{3},\tau \left(x\right)\right)$ of (21) such that (ϕ(0),$c_{1}\left(0\right),c_{2}\left(0\right),\tau \left(0\right))=\left(V,L_{1},L_{2},0\right)$ and $\left(\phi \left(a\right),c_{1}\left(a\right),c_{2}\left(a\right),\tau \left(a\right)\right)=\left(\phi^{\left[a,l\right]},c_{1}^{\left[a,l\right]},c_{2}^{\left[a,l\right]},a\right),$ where $\phi^{\left[a,l\right]}$, $c_{1}^{\left[a,l\right]}$ and $c_{2}^{\left[a,l\right]}$ are given in Proposition 2. It is given by*

(22) $$\begin{array}{c} \begin{array}{cc} \phi (x)= & V-\frac{T^{c}}{zz_{3}T^{m}}\ln\left(1+\frac{z_{3}T^{m}H\left(x\right)}{(z-z_{3})L}\right),\\ c_{1}\left(x\right)= & \frac{L_{1}J_{2}-L_{2}J_{1}}{J_{1}+J_{2}}{\left(1+\frac{z_{3}T^{m}H\left(x\right)}{(z-z_{3})L}\right)}^{\frac{T^{c}}{z_{3}T^{m}}}+\frac{LJ_{1}}{J_{1}+J_{2}}\left(1+\frac{z_{3}T^{m}H\left(x\right)}{(z-z_{3})L}\right),\\ c_{2}\left(x\right)= & \frac{L_{2}J_{1}-L_{1}J_{2}}{J_{1}+J_{2}}{\left(1+\frac{z_{3}T^{m}H\left(x\right)}{(z-z_{3})L}\right)}^{\frac{T^{c}}{z_{3}T^{m}}}+\frac{LJ_{2}}{J_{1}+J_{2}}\left(1+\frac{z_{3}T^{m}H\left(x\right)}{(z-z_{3})L}\right),\\ \tau (x)= & x, \end{array} \end{array}$$

*where $J_{1},\;J_{2}$ and $J_{3}$ are uniquely determined as*

(23) $$\begin{array}{c} \begin{array}{cc} J_{1}= & A_{1}B_{1}\frac{L_{1}-c_{1}^{\left[a,l\right]}e^{z(\phi^{\left[a,l\right]}-V)}}{H(a)},\;J_{2}=A_{1}B_{1}\frac{L_{2}-c_{2}^{\left[a,l\right]}e^{z(\phi^{\left[a,l\right]}-V)}}{H(a)},\\ J_{3}= & -\frac{z}{z_{3}}A_{1}\frac{\ln L-\ln C^{\left[a,l\right]}e^{z_{3}\left(\phi^{\left[a,l\right]}-V\right)}}{H(a)}, \end{array} \end{array}$$

*where $A_{1}=\frac{L-C^{\left[a,l\right]}}{\ln L-\ln C^{\left[a,l\right]}}$ and $B_{1}=\frac{\ln L-\ln C^{\left[a,l\right]}e^{z(\phi^{\left[a,l\right]}-V)}}{L-C^{\left[a,l\right]}e^{z(\phi^{\left[a,l\right]}-V)}}$*

**Proof.**  Adding the second equation to the third one in (21), one has

$$\begin{array}{c} {\dot{c}}_{1}+{\dot{c}}_{2}=\frac{z_{3}}{\left(z-z_{3}\right)h\left(\tau \right)}T^{m}, \end{array}$$

which gives

(24) $$\begin{array}{c} c_{1}\left(x\right)+c_{2}\left(x\right)=L+\frac{z_{3}}{z-z_{3}}T^{m}H\left(x\right). \end{array}$$

Substituting (24) into the second equation in (21) to get

$$\begin{array}{cc} {\dot{c}}_{1}= & \frac{T^{c}}{\left(z-z_{3}\right)L+z_{3}T^{m}H\left(x\right)}\frac{c_{1}}{h(\tau )}-\frac{J_{1}}{h(\tau )}. \end{array}$$

By the variation of constants formula, we obtain

$$\begin{array}{cc} c_{1}\left(x\right)= & \frac{L_{1}J_{2}-L_{2}J_{1}}{J_{1}+J_{2}}{\left(1+\frac{z_{3}T^{m}H\left(x\right)}{(z-z_{3})L}\right)}^{\frac{T^{c}}{z_{3}T^{m}}}+\frac{LJ_{1}}{J_{1}+J_{2}}\left(1+\frac{z_{3}T^{m}H\left(x\right)}{(z-z_{3})L}\right). \end{array}$$

Similarly, $c_{2}\left(x\right)$ can be obtained.

Substituting (24) into the first equation in (21) to get

$$\dot{\phi }=-\frac{T^{c}}{z\left(z-z_{3}\right)\left(L+\frac{z_{3}}{z-z_{3}}T^{m}H\left(x\right)\right)h\left(\tau \right)}.$$

The solution is $\phi \left(x\right)=\phi^{L}-\frac{T^{c}}{zz_{3}T^{m}}\ln\left(1+\frac{z_{3}T^{m}}{(z-z_{3})L}H\left(x\right)\right).$ Evaluating $c_{1}\left(x\right)$, $c_{2}\left(x\right)$ and ϕ(x) at x=a yield the formulas for $J_{1},\;J_{2}$ and $J_{3}$. □

The slow orbit

(25) $$\begin{array}{c} \Lambda_{l}\left(x\right)=\left(\phi \left(x\right),u\left(x\right),c_{1}\left(x\right),c_{2}\left(x\right),c_{3}\left(x\right),J_{1},J_{2},J_{3},\tau \left(x\right)\right) \end{array}$$

given in Lemma 2 connects $\omega (N_{r}^{0})$ and $\alpha (N_{l}^{a})$. Let ${\bar{M}}_{r}^{0}$ (resp., ${\bar{M}}_{l}^{a}$) be the forward (resp., backward) image of $\omega (N_{r}^{0})$ (resp., $\alpha (N_{l}^{a})$) under the slow flow (21). One has the following result (the proof follows exactly the same line as Proposition 3.7 in Section 3.1.2 of [43]).

**Proposition** **3.** *On the nine-dimensional slow manifold $S_{l}$, ${\bar{M}}_{r}^{0}$ and ${\bar{M}}_{l}^{a}$ intersect transversally along the unique orbit $\Lambda_{l}\left(x\right)$ given in (25).*

#### 2.1.2. Singular Orbit on [a,b] with $Q\left(x\right)=Q_{0}$

Following (13), we let

$$B_{b}=\left\{\left(\phi^{\left[b\right]},u,c_{1}^{\left[b\right]},c_{2}^{\left[b\right]},c_{3}^{\left[b\right]},J_{1},J_{2},J_{3},b\right)\in R^{9}:u,J_{1},J_{2},J_{3}\;\mathrm{arbitrary}\right\},$$

and construct a singular orbit on [a,b] connecting $B_{a}$ to $B_{b}$.

**Limiting fast dynamics and boundary layers on [a,b]** By a similar argument as in Section 2.1.1, one has the slow manifold

$$Z_{m}=\left\{u=0,\;z\left(c_{1}+c_{2}\right)+z_{3}c_{3}+Q_{0}=0\right\},$$

and the corresponding limiting fast system,

(26) $$\begin{array}{c} \begin{array}{cc} \phi^{\prime }= & u,\;u^{\prime }=-zc_{1}-zc_{2}-z_{3}c_{3}-Q_{0},\;c_{1}^{\prime }=-zc_{1}u,\\ c_{2}^{\prime }= & -zc_{2}u,\;c_{3}^{\prime }=-z_{3}c_{3}u,\;J_{1}^{\prime }=J_{2}^{\prime }=J_{3}^{\prime }=0,\;\tau^{\prime }=0. \end{array} \end{array}$$

For system (26), similar argument shows that the slow manifold $Z_{m}$ is normally hyperbolic. We denote the stable (resp. unstable) manifold of $Z_{m}$ by $W^{s}\left(Z_{m}\right)$ (resp. $W^{u}\left(Z_{m}\right)$). Let $M_{m}^{a}$ be the collection of orbits from $B_{a}$ in forward time under the flow of system (26) and $M_{m}^{b}$ be the collection of orbits from $B_{b}$ in backward time under the flow of system (26). Then, for a singular orbit connecting $B_{a}$ to $B_{b}$, the boundary layer $\Gamma_{m}^{a}$ at x=a must lie in $N_{m}^{a}=M_{m}^{a}\cap W^{s}\left(Z_{m}\right)$ and the boundary layer $\Gamma_{m}^{b}$ at x=b must lie in $N_{m}^{b}=M_{m}^{b}\cap W^{u}\left(Z_{m}\right)$. In this section, we will determine the boundary layers $N_{m}^{a}$ and $N_{m}^{b}$, and their landing points $\omega (N_{m}^{a})$ and $\alpha (N_{m}^{b})$ on the slow manifold $Z_{m}$. The regular layer, determined by the limiting slow system, will lie in $Z_{m}$ and connect the landing points $\omega (N_{m}^{a})$ at x=a and $\alpha (N_{m}^{b})$ at x=b.

Similarly, we have the following result.

**Proposition** **4.**

(i) *System (26) has the following eight integrals,*
  $$\begin{array}{cc} H_{1}= & \ln c_{1}+z\phi ,\;H_{2}=\ln c_{2}+z\phi ,\;H_{3}=\ln c_{3}+z_{3}\phi ,\\ H_{4}= & \frac{u^{2}}{2}-c_{1}-c_{2}-c_{3}+Q_{0}\phi ,\;H_{5}=J_{1},\;H_{6}=J_{2},\;H_{7}=J_{3},\;H_{8}=\tau . \end{array}$$
(ii) *The stable manifold $W^{s}\left(Z_{m}\right)$ intersects $B_{a}$ transversally at points $\left(\phi^{\left[a\right]},u_{m}\left(a\right),c_{1}^{\left[a\right]},c_{2}^{\left[a\right]},c_{3}^{\left[a\right]},J_{1},J_{2},J_{3},a\right),$ and the ω-limit set of $N_{m}^{a}=M_{m}^{a}⋂W^{s}\left(Z_{m}\right)$ is*
  $$\omega \left(N_{m}^{a}\right)=\left\{(\phi^{\left[a,m\right]},0,c_{1}^{\left[a,m\right]},c_{2}^{\left[a,m\right]},c_{3}^{\left[a,m\right]},J_{1},J_{2},J_{3},a)\right\},$$
  *where $J_{i}$ for i=1,2,3 are arbitrary, and $\phi^{\left[a,m\right]}$ is the unique solution of*
  (27) $$\begin{array}{c} zc_{1}^{\left[a\right]}e^{z(\phi^{\left[a\right]}-\phi )}+zc_{2}^{\left[a\right]}e^{z(\phi^{\left[a\right]}-\phi )}+z_{3}c_{3}^{\left[a\right]}e^{z_{3}\left(\phi^{\left[a\right]}-\phi \right)}+Q_{0}=0, \end{array}$$
  *where $c_{k}^{\left[a,m\right]}=c_{k}^{\left[a\right]}e^{z(\phi^{\left[a\right]}-\phi^{\left[a,m\right]})},\;c_{3}^{\left[a,m\right]}=c_{3}^{\left[a\right]}e^{z_{3}\left(\phi^{\left[a\right]}-\phi^{\left[a,m\right]}\right)}$ and*
  (28) $$\begin{array}{cc} u_{m}\left(a\right)= & sgn\left(\phi^{\left[a,m\right]}-\phi^{\left[a\right]}\right)\sqrt{K_{m}^{a}}, \end{array}$$
  *where $K_{m}^{a}=2C^{\left[a\right]}\left(1-e^{z(\phi^{\left[a\right]}-\phi^{\left[a,m\right]})}\right)+2c_{3}^{\left[a\right]}\left(1-e^{z_{3}\left(\phi^{\left[a\right]}-\phi^{\left[a,m\right]}\right)}\right)-2Q_{0}\left(\phi^{\left[a\right]}-\phi^{\left[a,m\right]}\right).$*
(iii) *The unstable manifold $W^{u}\left(Z_{m}\right)$ intersects $B_{b}$ transversally at points $\left(\phi^{\left[b\right]},u_{m}\left(b\right),c_{1}^{\left[b\right]},c_{2}^{\left[b\right]},c_{3}^{\left[b\right]},J_{1},J_{2},J_{3},b\right),$ and the α-limit set of $N_{m}^{b}=M_{m}^{b}⋂W^{u}\left(Z_{m}\right)$ is*
  $$\alpha \left(N_{m}^{b}\right)=\left\{(\phi^{\left[b,m\right]},0,c_{1}^{\left[b,m\right]},c_{2}^{\left[b,m\right]},c_{3}^{\left[b,m\right]},J_{1},J_{2},J_{3},b)\right\},$$
  *where $J_{i}$ for i=1,2,3 are arbitrary, and $\phi^{\left[b,m\right]}$ is the unique solution of*
  (29) $$\begin{array}{c} zc_{1}^{\left[b\right]}e^{z(\phi^{\left[b\right]}-\phi )}+zc_{2}^{\left[b\right]}e^{z(\phi^{\left[b\right]}-\phi )}+z_{3}c_{3}^{\left[b\right]}e^{z_{3}\left(\phi^{\left[b\right]}-\phi \right)}+Q_{0}=0, \end{array}$$
  *where $c_{k}^{\left[b,m\right]}=c_{k}^{\left[b\right]}e^{z(\phi^{\left[b\right]}-\phi^{\left[b,m\right]})},\;c_{3}^{\left[b,m\right]}=c_{3}^{\left[b\right]}e^{z_{3}\left(\phi^{\left[b\right]}-\phi^{\left[b,m\right]}\right)}$ and*
  (30) $$\begin{array}{cc} u_{m}\left(b\right)= & sgn\left(\phi^{\left[b\right]}-\phi^{\left[b,m\right]}\right)\sqrt{K_{m}^{b}}, \end{array}$$
  *where $K_{m}^{b}=2C^{\left[b\right]}\left(1-e^{z(\phi^{\left[b\right]}-\phi^{\left[b,m\right]})}\right)+2c_{3}^{\left[b\right]}\left(1-e^{z_{3}\left(\phi^{\left[b\right]}-\phi^{\left[b,m\right]}\right)}\right)-2Q_{0}\left(\phi^{\left[b\right]}-\phi^{\left[b,m\right]}\right).$*
(iv) *The boundary layer $\Gamma_{m}^{a}$ at x=a is determined up to $\left(J_{1},J_{2},J_{3}\right)$ as follows: the ϕ-component satisfies the Hamiltonian system*
  $$\phi^{\prime \prime }+zc_{1}^{\left[a\right]}e^{z(\phi^{\left[a\right]}-\phi )}+zc_{2}^{\left[a\right]}e^{z(\phi^{\left[a\right]}-\phi )}+z_{3}c_{3}^{\left[a\right]}e^{z_{3}\left(\phi^{\left[a\right]}-\phi \right)}+Q_{0}=0,$$
  *together with $\phi \left(a\right)=\phi^{\left[a\right]}$ and $\phi \left(\xi \right)\to \phi^{\left[a,m\right]}$ as $\xi \to \infty ,\;u\left(\xi \right)=\phi^{\prime }\left(\xi \right),$ and*
  $$c_{1}\left(\xi \right)=c_{1}^{\left[a\right]}e^{z(\phi^{\left[a\right]}-\phi \left(\xi \right))},\;c_{2}\left(\xi \right)=c_{2}^{\left[a\right]}e^{z(\phi^{\left[a\right]}-\phi \left(\xi \right))},\;c_{3}\left(\xi \right)=c_{3}^{\left[a\right]}e^{z_{3}\left(\phi^{\left[a\right]}-\phi \left(\xi \right)\right)}.$$
*Similarly, the boundary layer $\Gamma_{m}^{b}$ at x=b is determined in the following way: the ϕ-component satisfies the Hamiltonian system*
  $$\phi^{\prime \prime }+zc_{1}^{\left[b\right]}e^{z(\phi^{\left[b\right]}-\phi )}+zc_{2}^{\left[b\right]}e^{z(\phi^{\left[b\right]}-\phi )}+z_{3}c_{3}^{\left[b\right]}e^{z_{3}\left(\phi^{\left[b\right]}-\phi \right)}+Q_{0}=0,$$
  *together with $\phi \left(b\right)=\phi^{\left[b\right]}$ and $\phi \left(\xi \right)\to \phi^{\left[b,m\right]}$ as $\xi \to -\infty ,\;u\left(\xi \right)=\phi^{\prime }\left(\xi \right),$ and*
  $$c_{1}\left(\xi \right)=c_{1}^{\left[b\right]}e^{z(\phi^{\left[b\right]}-\phi \left(\xi \right))},\;c_{2}\left(\xi \right)=c_{2}^{\left[b\right]}e^{z(\phi^{\left[b\right]}-\phi \left(\xi \right))},\;c_{3}\left(\xi \right)=c_{3}^{\left[b\right]}e^{z_{3}\left(\phi^{\left[b\right]}-\phi \left(\xi \right)\right)}.$$

**Limiting slow dynamics and regular layers on [a,b]** We now turn to the study of the flow in the vicinity of the slow manifold $Z_{m}$. Following a similar argument as that in Section 2.1.1, we make a scaling u=εp and $-zc_{1}-zc_{2}-z_{3}c_{3}-Q_{0}=\varepsilon q.$ System (10) becomes

$$\begin{array}{c} \begin{array}{cc} \dot{\phi }= & p,\;\varepsilon \dot{p}=q-\varepsilon \frac{h^{\prime }\left(\tau \right)}{h(\tau )}p,\;\varepsilon \dot{q}=\left(z\left(z-z_{3}\right)\left(c_{1}+c_{2}\right)-z_{3}Q_{0}-\varepsilon z_{3}q\right)p+\frac{T^{c}}{h(\tau )},\\ {\dot{c}}_{1}= & -zc_{1}p-\frac{J_{1}}{h(\tau )},\;{\dot{c}}_{2}=-zc_{2}p-\frac{J_{2}}{h(\tau )},\;{\dot{J}}_{k}=0,\;\dot{\tau }=1. \end{array} \end{array}$$

It is again a singular perturbation problem and its limiting slow system is

(31) $$\begin{array}{c} \begin{array}{cc} \dot{\phi }= & p,\;q=0,\;p=-\frac{T^{c}}{\left(z\left(z-z_{3}\right)\left(c_{1}+c_{2}\right)-z_{3}Q_{0}\right)h\left(\tau \right)},\\ {\dot{c}}_{1}= & -zc_{1}p-\frac{J_{1}}{h(\tau )},\;{\dot{c}}_{2}=-zc_{2}p-\frac{J_{2}}{h(\tau )},\;{\dot{J}}_{k}=0,\;\dot{\tau }=1. \end{array} \end{array}$$

For system (31), the slow manifold is

$$S_{m}=\left\{q=0,\;p=-\frac{T^{c}}{\left(z\left(z-z_{3}\right)\left(c_{1}+c_{2}\right)-z_{3}Q_{0}\right)h\left(\tau \right)}\right\}.$$

Therefore, the limiting slow system on $S_{m}$ is given by

(32) $$\begin{array}{c} \begin{array}{cc} \dot{\phi }= & -\frac{T^{c}}{\left(z\left(z-z_{3}\right)\left(c_{1}+c_{2}\right)-z_{3}Q_{0}\right)h\left(\tau \right)},\\ {\dot{c}}_{1}= & \frac{T^{c}}{\left(z\left(z-z_{3}\right)\left(c_{1}+c_{2}\right)-z_{3}Q_{0}\right)h\left(\tau \right)}zc_{1}-\frac{J_{1}}{h(\tau )},\\ {\dot{c}}_{2}= & \frac{T^{c}}{\left(z\left(z-z_{3}\right)\left(c_{1}+c_{2}\right)-z_{3}Q_{0}\right)h\left(\tau \right)}zc_{2}-\frac{J_{2}}{h(\tau )},\\ {\dot{J}}_{k}= & 0,\;\dot{\tau }=1. \end{array} \end{array}$$

Note that, on $Z_{m},$ one has $z\left(c_{1}+c_{2}\right)+z_{3}c_{3}+Q_{0}=0.$ It follows that

$$z\left(z-z_{3}\right)\left(c_{1}+c_{2}\right)-z_{3}Q_{0}=z^{2}\left(c_{1}+c_{2}\right)+z_{3}^{2}c_{3}>0.$$

Since h(τ)>0 and $z\left(z-z_{3}\right)\left(c_{1}+c_{2}\right)-z_{3}Q_{0}>0,$ system (32) has the same phase portrait as that of the following system obtained by multiplying $\left(z\left(z-z_{1}\right)\left(c_{2}+c_{3}\right)-z_{1}Q_{0}\right)h\left(\tau \right)$ on the right-hand side of system (32):

(33) $$\begin{array}{c} \begin{array}{cc} \frac{d\phi }{dy}= & -T^{c},\;\frac{dc_{1}}{dy}=T^{c}zc_{1}-J_{1}\left(z\left(z-z_{3}\right)\left(c_{1}+c_{2}\right)-z_{3}Q_{0}\right),\\ \frac{dc_{2}}{dy}= & T^{c}zc_{2}-J_{2}\left(z\left(z-z_{3}\right)\left(c_{1}+c_{2}\right)-z_{3}Q_{0}\right),\\ \frac{dJ_{k}}{dy}= & 0,\;\frac{d\tau }{dy}=h\left(\tau \right)\left(z\left(z-z_{3}\right)\left(c_{1}+c_{2}\right)-z_{3}Q_{0}\right). \end{array} \end{array}$$

**Lemma** **3.** *There is a unique solution $\left(\phi \left(x\right),c_{1}\left(x\right),c_{2}\left(x\right),J_{1},J_{2},J_{3},\tau \left(x\right)\right)$ of (33) such that (ϕ(a),$c_{1}\left(a\right),c_{2}\left(a\right),\tau \left(a\right))=\left(\phi^{\left[a,m\right]},c_{1}^{\left[a,m\right]},c_{2}^{\left[a,m\right]},a\right)$ and $\left(\phi \left(b\right),c_{1}\left(b\right),c_{2}\left(b\right),\tau \left(b\right)\right)=(\phi^{\left[b,m\right]},c_{1}^{\left[b,m\right]},c_{2}^{\left[b,m\right]},$b), where $\phi^{\left[a,m\right]}$, $\phi^{\left[b,m\right]}$, $c_{1}^{\left[a,m\right]}$, $c_{1}^{\left[b,m\right]}$, $c_{2}^{\left[a,m\right]}$ and $c_{2}^{\left[b,m\right]}$ are given in Proposition 4. It is given by*

(34) $$\begin{array}{c} \begin{array}{cc} \; & \phi \left(y\right)=\phi^{\left[a,m\right]}-T^{c}y,\;c_{1}\left(y\right)=\frac{J_{2}c_{1}^{\left[a,m\right]}-J_{1}c_{2}^{\left[a,m\right]}}{J_{1}+J_{2}}e^{zT^{c}y}-J_{1}\cdot A_{3}\left(y\right),\\ \; & c_{2}\left(y\right)=\frac{J_{1}c_{2}^{\left[a,m\right]}-J_{2}c_{1}^{\left[a,m\right]}}{J_{1}+J_{2}}e^{zT^{c}y}-J_{2}\cdot A_{3}\left(y\right),\\ \; & \int_{a}^{\tau }\frac{1}{h(s)}ds=\frac{z-z_{3}}{z_{3}T^{m}}\left(e^{zz_{3}T^{m}y}-1\right)\left(C^{\left[a,m\right]}+\frac{\left(J_{1}+J_{2}\right)Q_{0}}{zT^{m}}\right)-\frac{T^{c}}{T^{m}}Q_{0}y, \end{array} \end{array}$$

*where $A_{3}\left(y\right)=\frac{Q_{0}}{zT^{m}}\left(1-e^{zz_{3}T^{m}y}\right)-\frac{C^{\left[a,m\right]}}{J_{1}+J_{2}}e^{zz_{3}T^{m}y},$ and $J_{1},\;J_{2}$ and $J_{3}$ are uniquely determined as, for some $y_{0}>0,$*

(35) $$\begin{array}{c} \begin{array}{cc} \; & \phi^{\left[b,m\right]}=\phi^{\left[a,m\right]}-T^{c}y_{0},\;c_{1}^{\left[b,m\right]}=\frac{J_{2}c_{1}^{\left[a,m\right]}-J_{1}c_{2}^{\left[a,m\right]}}{J_{1}+J_{2}}e^{zT^{c}y_{0}}-J_{1}\cdot A_{3}\left(y_{0}\right),\\ \; & c_{2}^{\left[b,m\right]}=\frac{J_{1}c_{2}^{\left[a,m\right]}-J_{2}c_{1}^{\left[a,m\right]}}{J_{1}+J_{2}}e^{zT^{c}y_{0}}-J_{2}\cdot A_{3}\left(y_{0}\right),\\ \; & T^{m}=\frac{\left(z-z_{3}\right)\left(C^{\left[b,m\right]}-C^{\left[a,m\right]}\right)+z_{3}Q_{0}\left(\phi^{\left[b,m\right]}-\phi^{\left[a,m\right]}\right)}{z_{3}\left(H\left(b\right)-H\left(a\right)\right)}. \end{array} \end{array}$$

**Remark** **2.** *The proof is similar as that of Lemma 2. As for the system (35), note that we are looking for solutions to reach $\alpha (N_{m}^{a})$; that is, whenever τ(y)=b, we require $\phi \left(y\right)=\phi^{\left[b,m\right]},\;c_{2}\left(y\right)=c_{2}^{\left[b,m\right]}$ and $c_{3}\left(y\right)=c_{3}^{\left[b,m\right]}$. Assume $\tau (y_{0})=b$ for some $y_{0}>0$. Then, $\phi \left(y_{0}\right)=\phi^{\left[b,m\right]},$$c_{2}\left(y_{0}\right)=c_{2}^{\left[b,m\right]}$ and $c_{3}\left(y_{0}\right)=c_{3}^{\left[b,m\right]}$. Evaluating system (34) at $y=y_{0},$ by a careful calculation, one has system (35).*

It follows that the regular layer solution $\Lambda_{m}$ on [a,b] is given by (34) with $J_{1},J_{2}$ and $J_{3}$ determined by (35). Together with the boundary layers $\Gamma_{m}^{a}$ and $\Gamma_{m}^{b}$ described in statement (iv) of Proposition 4, this gives the singular orbit on the interval [a,b].

#### 2.1.3. Singular Orbit on [b,1] with Q(x)=0

Th construction of singular orbits over [b,1] from $B_{b}$ to $B_{R}$ is virtually the same as the construction of singular orbits on [0,a] studied in Section 2.1.1. Instead of repeating the same process, we will state only the results for later use.

**Limiting fast dynamics and boundary layers on [b,1]** The limiting fast system reads

(36) $$\begin{array}{c} \begin{array}{cc} \phi^{\prime }= & u,\;u^{\prime }=-zc_{1}-zc_{2}-z_{3}c_{3},\;c_{1}^{\prime }=-zc_{1}u,\\ c_{2}^{\prime }= & -zc_{2}u,\;c_{3}^{\prime }=-z_{3}c_{3}u,\;J_{1}^{\prime }=J_{2}^{\prime }=J_{3}^{\prime }=0,\;\tau^{\prime }=0. \end{array} \end{array}$$

The slow manifold is $Z_{r}=\left\{u=0,z\left(c_{1}+c_{2}\right)+z_{3}c_{3}=0\right\},$ which consists of the equilibria of system (36) and is normally hyperbolic with a eight-dimensional center-stable manifold $W^{s}\left(Z_{r}\right)$ and a eight-dimensional center-unstable manifold $W^{u}\left(Z_{r}\right)$. For the boundary layers, one has the following proposition.

**Proposition** **5.** *Under the condition (15), one has*

(i) *System (36) has the following integrals:*
  $$\begin{array}{cc} H_{1}= & \ln c_{1}+z\phi ,\;H_{2}=\ln c_{2}+z\phi ,\;H_{3}=\ln c_{3}+z_{3}\phi ,\\ H_{4}= & \frac{u^{2}}{2}-c_{1}-c_{2}-c_{3},\;H_{5}=J_{1},\;H_{6}=J_{2},\;H_{7}=J_{3},\;H_{8}=\tau . \end{array}$$
(ii) *The stable manifold $W^{s}\left(Z_{r}\right)$ intersects $B_{b}$ transversally at points $\left(\phi^{\left[b\right]},u_{r}\left(b\right),c_{1}^{\left[b\right]},c_{2}^{\left[b\right]},c_{3}^{\left[b\right]},J_{1},J_{2},J_{3},b\right),$ and the ω-limit set of $N_{r}^{b}=M_{r}^{b}⋂W^{s}\left(Z_{r}\right)$ is*
  $$\omega \left(N_{r}^{b}\right)=\left\{(\phi^{\left[b,r\right]},0,c_{1}^{\left[b,r\right]},c_{2}^{\left[b,r\right]},c_{3}^{\left[b,r\right]},J_{1},J_{2},J_{3},b)\right\},$$
  *where $J_{i}$ for i=1,2,3 are arbitrary, and*
  $$\begin{array}{cc} \phi^{\left[b,r\right]}= & \phi^{b}-\frac{1}{z-z_{3}}\ln\frac{-z_{3}c_{3}^{\left[b\right]}}{zC^{\left[b\right]}},\;c_{k}^{\left[b,r\right]}=c_{k}^{\left[b\right]}{\left(\frac{-z_{3}c_{3}^{\left[b\right]}}{zC^{\left[b\right]}}\right)}^{\frac{z}{z-z_{3}}},\;c_{3}^{\left[b,r\right]}=c_{3}^{\left[b\right]}{\left(\frac{-z_{3}c_{3}^{\left[b\right]}}{zC^{\left[b\right]}}\right)}^{\frac{z_{3}}{z-z_{3}}} \end{array}$$
  *and*
  (37) $$\begin{array}{c} \begin{array}{cc} \; & u_{r}\left(b\right)=sgn\left(\phi^{\left[b\right]}-\phi^{\left[b,r\right]}\right)\sqrt{2C^{\left[b\right]}\left(1-e^{z(\phi^{\left[b\right]}-\phi^{\left[b,r\right]})}\right)+2c_{3}^{\left[b\right]}\left(1-e^{z_{3}\left(\phi^{\left[b\right]}-\phi^{\left[b,r\right]}\right)}\right)}. \end{array} \end{array}$$
(iii) *The unstable manifold $W^{u}\left(Z_{r}\right)$ intersects $B_{R}$ transversally at points $\left(0,u_{l}^{1},R_{1},R_{2},R_{3},J_{1},J_{2},J_{3},1\right),$ and the α-limit set of $N_{l}^{1}=M_{l}^{1}⋂W^{u}\left(Z_{r}\right)$ is*
  $$\alpha \left(N_{r}^{1}\right)=\left\{(\phi^{R},0,c_{1}^{R},c_{2}^{R},c_{3}^{R},J_{1},J_{2},J_{3},1)\right\},$$
  *where $J_{i}$ for i=1,2,3 are arbitrary, and*
  $$\begin{array}{cc} \phi^{R}= & 0,\;c_{1}^{R}=R_{1},\;c_{2}^{R}=R_{2},\;c_{3}^{R}=R_{3},\;u_{l}^{1}=0. \end{array}$$
(iv) *The boundary layer $\Gamma_{r}^{b}$ at x=b is determined up to $\left(J_{1},J_{2},J_{3}\right)$ as follows: the ϕ-component satisfies the Hamiltonian system*
  $$\phi^{\prime \prime }+zc_{1}^{\left[b\right]}e^{z(\phi^{\left[b\right]}-\phi )}+zc_{2}^{\left[b\right]}e^{z(\phi^{\left[b\right]}-\phi )}+z_{3}c_{3}^{b}e^{z_{3}\left(\phi^{\left[b\right]}-\phi \right)}=0,$$
  *together with $\phi \left(b\right)=\phi^{\left[b\right]}$ and $\phi \left(\xi \right)\to \phi^{\left[b,r\right]}$ as $\xi \to \infty ,\;u\left(\xi \right)=\phi^{\prime }\left(\xi \right),$ and*
  $$c_{1}\left(\xi \right)=c_{1}^{\left[b\right]}e^{z(\phi^{\left[b\right]}-\phi \left(\xi \right))},\;c_{2}\left(\xi \right)=c_{2}^{\left[b\right]}e^{z(\phi^{\left[b\right]}-\phi \left(\xi \right))},\;c_{3}\left(\xi \right)=c_{3}^{\left[b\right]}e^{z_{3}\left(\phi^{\left[b\right]}-\phi \left(\xi \right)\right)}.$$
*Similarly, the boundary layer $\Gamma_{r}^{1}$ at x=1 is determined in the following way: the ϕ-component satisfies the Hamiltonian system*
  $$\phi^{\prime \prime }+zR_{1}e^{-z\phi }+zR_{2}e^{-z\phi }+z_{3}R_{3}e^{-z_{3}\phi }=0,$$
  *together with ϕ(1)=0 and $\phi \left(\xi \right)\to \phi^{R}$ as $\xi \to -\infty ,\;u\left(\xi \right)=\phi^{\prime }\left(\xi \right),$ and*
  $$c_{1}\left(\xi \right)=R_{1}e^{-z\phi (\xi )},\;c_{2}\left(\xi \right)=R_{2}e^{-z\phi (\xi )},\;c_{3}\left(\xi \right)=R_{3}e^{-z_{3}\phi \left(\xi \right)}.$$

**Limiting slow dynamics and regular layers on [b,1]** We now examine the existence of the regular layer on $Z_{r}$ that connects $\omega (N_{r}^{b})$ and $\alpha (N_{r}^{1})$. It follows from exactly the same analysis as that in Section 2.1.1, the limiting slow dynamics reads

(38) $$\begin{array}{c} \begin{array}{cc} \dot{\phi }= & -\frac{T^{c}}{z\left(z-z_{3}\right)\left(c_{1}+c_{2}\right)h\left(\tau \right)},\;{\dot{c}}_{1}=\frac{T^{c}}{\left(z-z_{3}\right)\left(c_{1}+c_{2}\right)h\left(\tau \right)}c_{1}-\frac{J_{1}}{h(\tau )},\\ {\dot{c}}_{2}= & \frac{T^{c}}{\left(z-z_{3}\right)\left(c_{1}+c_{2}\right)h\left(\tau \right)}c_{2}-\frac{J_{2}}{h(\tau )},\;{\dot{J}}_{k}=0,\;\dot{\tau }=1. \end{array} \end{array}$$

For system (38), one has

**Lemma** **4.** *There is a unique solution $\left(\phi \left(x\right),c_{1}\left(x\right),c_{2}\left(x\right),J_{1},J_{2},J_{3},\tau \left(x\right)\right)$ of (38) such that $\left(\phi \left(b\right),c_{1}\left(b\right),c_{2}\left(b\right),\tau \left(b\right)\right)=\left(\phi^{\left[b,r\right]},c_{1}^{\left[b,r\right]},c_{2}^{\left[b,r\right]},b\right)$ and $\left(\phi \left(1\right),c_{1}\left(1\right),c_{2}\left(1\right),\tau \left(1\right)\right)=\left(0,R_{1},R_{2},1\right),$ where $\phi^{\left[b,r\right]}$, $c_{1}^{\left[b,r\right]}$ and $c_{2}^{\left[b,r\right]}$ are given in Proposition 5. It is given by*

$$\begin{array}{c} \begin{array}{cc} \phi (x)= & \phi^{\left[b,r\right]}-\frac{T^{c}\ln M_{1}\left(x\right)}{zz_{3}T^{m}},\;c_{1}\left(x\right)=\frac{c_{1}^{\left[b,r\right]}J_{2}-c_{2}^{\left[b,r\right]}J_{1}}{J_{1}+J_{2}}{\left(M_{1}\left(x\right)\right)}^{\frac{T^{c}}{z_{3}T^{m}}}+\frac{C^{\left[b,r\right]}J_{1}}{J_{1}+J_{2}}M_{2}\left(x\right),\\ c_{2}\left(x\right)= & \frac{c_{2}^{\left[b,r\right]}J_{1}-c_{1}^{\left[b,r\right]}J_{2}}{J_{1}+J_{2}}{\left(M_{1}\left(x\right)\right)}^{\frac{T^{c}}{z_{3}T^{m}}}+\frac{C^{\left[b,r\right]}J_{2}}{J_{1}+J_{2}}M_{2}\left(x\right),\;\tau \left(x\right)=x, \end{array} \end{array}$$

*where $M_{1}\left(x\right)=1+\frac{z_{3}T^{m}\left(H\left(x\right)-H\left(b\right)\right)}{\left(z-z_{3}\right)C^{\left[b,r\right]}},\;M_{2}\left(x\right)=1+\frac{z_{1}T^{m}\left(H\left(x\right)-H\left(b\right)\right)}{\left(z-z_{3}\right)C^{\left[b,r\right]}},$ and $J_{1},\;J_{2}$ and $J_{3}$ are uniquely determined as*

(39) $$\begin{array}{c} \begin{array}{cc} J_{1}= & A_{2}B_{2}\frac{c_{1}^{\left[b,r\right]}-R_{1}e^{-z\phi^{\left[b,r\right]}}}{H(1)-H(b)},\;J_{2}=A_{2}B_{2}\frac{c_{2}^{\left[b,r\right]}-R_{2}e^{-z\phi^{\left[b,r\right]}}}{H(1)-H(b)},\\ J_{3}= & -\frac{z}{z_{3}}A_{2}\frac{\ln C^{\left[b,r\right]}-\ln Re^{-z_{3}\phi^{\left[b,r\right]}}}{H(1)-H(b)}, \end{array} \end{array}$$

*where $A_{2}=\frac{C^{\left[b,r\right]}-R}{\ln C^{\left[b,r\right]}-\ln R}$ and $B_{2}=\frac{\ln C^{\left[b,r\right]}-\ln Re^{-z\phi^{b,r}}}{C^{\left[b,r\right]}-Re^{-z\phi^{\left[b,r\right]}}}.$*

The regular solution $\Lambda_{r}$ that connects $\omega (N_{r}^{b})$ and $\alpha (N_{l}^{1})$, together with $\Gamma_{r}^{b}$ and $\Gamma_{l}^{1}$ in statement (iv) of Proposition 5 yields the singular orbit on [b,1].

#### 2.1.4. Matching and Singular Orbits on the Whole Interval [0,1]

**Figure 1:**
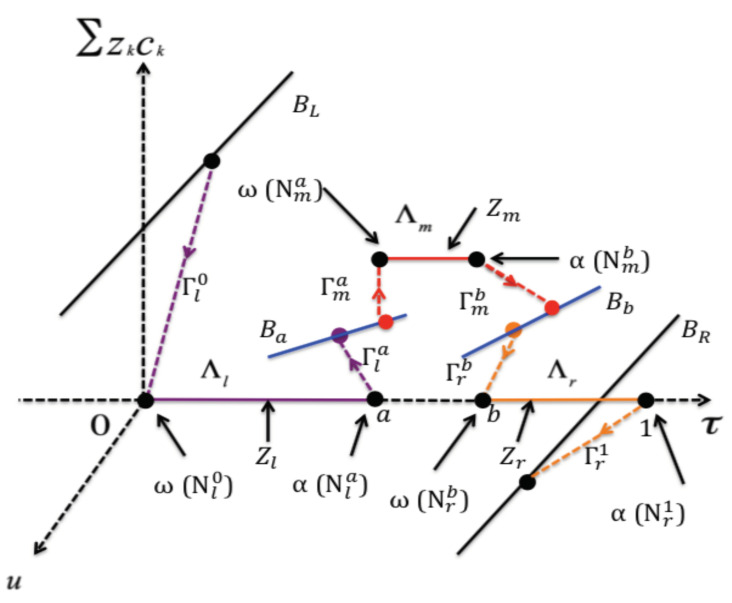
Schematic picture of a singular orbit projected to the space of $\left(u,\sum z_{k}c_{k},\tau \right)$ with Q(x) defined in (6).

A singular orbit over the whole interval [0,1] will be the union of the singular orbits constructed on each of the subintervals (see Figure 1). The matching conditions are $u_{l}\left(a\right)=u_{m}\left(a\right),$$u_{m}\left(b\right)=u_{r}\left(b\right)$, and $J_{1},\;J_{2}$ and $J_{3}$ have to be the same on all subintervals; that is, from (18), (23), (27)–(30), (35), (37) and (39),

(40) $$\begin{array}{c} \begin{array}{cc} \; & 0=C^{\left[a\right]}\left(e^{z(\phi^{\left[a\right]}-\phi^{\left[a,m\right]})}-e^{z(\phi^{\left[a\right]}-\phi^{\left[a,l\right]})}\right)+c_{3}^{\left[a\right]}\left(e^{z_{3}\left(\phi^{\left[a\right]}-\phi^{\left[a,m\right]}\right)}-e^{z_{3}\left(\phi^{\left[a\right]}-\phi^{\left[a,l\right]}\right)}\right)\\ & \;+Q_{0}\left(\phi^{\left[a\right]}-\phi^{\left[a,m\right]}\right),\\ \; & 0=C^{\left[b\right]}\left(e^{z(\phi^{\left[b\right]}-\phi^{\left[b,r\right]})}-e^{z(\phi^{\left[b\right]}-\phi^{\left[b,m\right]})}\right)+c_{3}^{\left[b\right]}\left(e^{z_{3}\left(\phi^{\left[b\right]}-\phi^{\left[b,r\right]}\right)}-e^{z_{3}\left(\phi^{\left[b\right]}-\phi^{\left[b,m\right]}\right)}\right)\\ & \;-Q_{0}\left(\phi^{\left[b\right]}-\phi^{\left[b,m\right]}\right),\\ \; & 0=zc_{1}^{\left[a\right]}e^{z(\phi^{\left[a\right]}-\phi^{\left[a,m\right]})}+zc_{2}^{\left[a\right]}e^{z(\phi^{\left[a\right]}-\phi^{\left[a,m\right]})}+z_{3}c_{3}^{\left[a\right]}e^{z_{3}\left(\phi^{\left[a\right]}-\phi^{\left[a,m\right]}\right)}+Q_{0},\\ \; & 0=zc_{1}^{\left[b\right]}e^{z(\phi^{\left[b\right]}-\phi^{\left[b,m\right]})}+zc_{2}^{\left[b\right]}e^{z(\phi^{\left[b\right]}-\phi^{\left[b,m\right]})}+z_{3}c_{3}^{\left[b\right]}e^{z_{3}\left(\phi^{\left[b\right]}-\phi^{\left[b,m\right]}\right)}+Q_{0},\\ \; & J_{1}=A_{1}B_{1}\frac{L_{1}-c_{1}^{\left[a,l\right]}e^{z(\phi^{a,l}-V)}}{H(a)}=A_{2}B_{2}\frac{c_{1}^{\left[b,r\right]}-R_{1}e^{-z\phi^{\left[b,r\right]}}}{H(1)-H(b)},\\ \; & J_{2}=A_{1}B_{1}\frac{L_{2}-c_{2}^{a[,l]}e^{z(\phi^{a,l}-V)}}{H(a)}=A_{2}B_{2}\frac{c_{2}^{\left[b,r\right]}-R_{2}e^{-z\phi^{\left[b,r\right]}}}{H(1)-H(b)},\\ \; & J_{3}=-\frac{z}{z_{3}}A_{1}\frac{\ln L-\ln C^{\left[a,l\right]}e^{z_{3}\left(\phi^{a,l}-V\right)}}{H(a)}=-\frac{z}{z_{3}}A_{2}\frac{\ln C^{\left[b,r\right]}-\ln Re^{-z_{3}\phi^{\left[b,r\right]}}}{H(1)-H(b)},\\ \; & \phi^{\left[b,m\right]}=\phi^{\left[a,m\right]}-T^{c}y_{0},\;c_{1}^{\left[b,m\right]}=\frac{J_{2}c_{1}^{\left[a,m\right]}-J_{1}c_{2}^{\left[a,m\right]}}{J_{1}+J_{2}}e^{zT^{c}y_{0}}-J_{1}\cdot A_{3}\left(y_{0}\right),\\ \; & c_{2}^{\left[b,m\right]}=\frac{J_{1}c_{2}^{\left[a,m\right]}-J_{2}c_{1}^{\left[a,m\right]}}{J_{1}+J_{2}}e^{zT^{c}y_{0}}-J_{2}\cdot A_{3}\left(y_{0}\right),\\ \; & T^{m}=\frac{\left(z-z_{3}\right)\left(C^{\left[b,m\right]}-C^{\left[a,m\right]}\right)+z_{3}Q_{0}\left(\phi^{\left[b,m\right]}-\phi^{\left[a,m\right]}\right)}{z_{3}\left(H\left(b\right)-H\left(a\right)\right)}. \end{array} \end{array}$$

where, for k=1,2,

(41) $$\begin{array}{c} \begin{array}{cc} \; & \phi^{L}=V,\;c_{1}^{L}=L_{1},\;c_{2}^{L}=L_{2},\;c_{3}^{L}=L_{3},\;\phi^{\left[a,l\right]}=\phi^{\left[a\right]}-\frac{1}{z-z_{3}}\ln\frac{-z_{3}c_{3}^{\left[a\right]}}{zC^{\left[a\right]}},\\ \; & c_{k}^{\left[a,l\right]}=c_{k}^{\left[a\right]}{\left(\frac{-z_{3}c_{3}^{\left[a\right]}}{zC^{\left[a\right]}}\right)}^{\frac{z}{z-z_{3}}},\;c_{3}^{\left[a,l\right]}=c_{3}^{\left[a\right]}{\left(\frac{-z_{3}c_{3}^{\left[a\right]}}{zC^{\left[a\right]}}\right)}^{\frac{z_{3}}{z-z_{3}}},\\ \; & c_{k}^{\left[b,r\right]}=c_{k}^{\left[b\right]}{\left(\frac{-z_{3}c_{3}^{\left[b\right]}}{zC^{\left[b\right]}}\right)}^{\frac{z}{z-z_{3}}},\;c_{3}^{b,r}=c_{3}^{\left[b\right]}{\left(\frac{-z_{3}c_{3}^{\left[b\right]}}{zC^{\left[b\right]}}\right)}^{\frac{z_{3}}{z-z_{3}}},\\ \; & \phi^{\left[b,r\right]}=\phi^{\left[b\right]}-\frac{1}{z-z_{3}}\ln\frac{-z_{3}c_{3}^{\left[b\right]}}{zC^{\left[b\right]}},\;\phi^{R}=0,\;c_{1}^{R}=R_{1},\;c_{2}^{R}=R_{2},\;c_{3}^{R}=R_{3},\\ \; & c_{k}^{\left[a,m\right]}=c_{k}^{\left[a\right]}e^{z(\phi^{\left[a\right]}-\phi^{\left[a,m\right]})},\;c_{3}^{\left[a,m\right]}=c_{3}^{\left[a\right]}e^{z_{3}\left(\phi^{\left[a\right]}-\phi^{\left[a,m\right]}\right)},\\ \; & c_{k}^{\left[b,m\right]}=c_{k}^{\left[b\right]}e^{z(\phi^{\left[b\right]}-\phi^{\left[b,m\right]})},\;c_{3}^{\left[b,m\right]}=c_{3}^{\left[b\right]}e^{z_{3}\left(\phi^{\left[b\right]}-\phi^{\left[b,m\right]}\right)},\\ \; & A_{1}=\frac{L-C^{\left[a,l\right]}}{\ln L-\ln C^{\left[a,l\right]}},\;B_{1}=\frac{\ln L-\ln C^{\left[a,l\right]}e^{z(\phi^{\left[a,l\right]}-V)}}{L-C^{\left[a,l\right]}e^{z(\phi^{\left[a,l\right]}-V)}},\;A_{2}=\frac{C^{\left[b,r\right]}-R}{\ln C^{\left[b,r\right]}-\ln R},\\ \; & B_{2}=\frac{\ln C^{\left[b,r\right]}-\ln Re^{-z\phi^{\left[b,r\right]}}}{C^{\left[b,r\right]}-Re^{-z\phi^{\left[b,r\right]}}},\;A_{3}\left(y\right)=\frac{Q_{0}}{zT^{m}}\left(1-e^{zz_{3}T^{m}y}\right)-\frac{C^{\left[a,m\right]}}{J_{1}+J_{2}}e^{zz_{3}T^{m}y}. \end{array} \end{array}$$

Recall that $\left(\phi^{\left[a\right]},c_{1}^{\left[a\right]},c_{2}^{\left[a\right]},c_{3}^{\left[a\right]}\right)$ and $\left(\phi^{\left[b\right]},c_{1}^{\left[b\right]},c_{2}^{\left[b\right]},c_{3}^{\left[b\right]}\right)$ are the unknown values preassigned at x=a and x=b, $J_{1},\;J_{2}$ and $J_{3}$ are the unknown values for the flux densities of the three ion species. There are also three auxiliary unknowns $\phi^{\left[a,m\right]},\;\phi^{\left[b,m\right]}$ and $y_{0}$ in (40). The total number of unknowns in (40) is fourteen, which matches the total number of equations.

A qualitative important question is whether the set of nonlinear Equation (40) (a governing system) has a unique solution. This can be studied through bifurcation analysis and numerical simulations. However, this is beyond the aim of this work.

#### 2.1.5. Existence of Solutions near the Singular Orbit

Note that any solution of the set of algebraic equations determines a singular orbit for the connection problem. Once a singular orbit is constructed, one can apply geometric singular perturbation theory to show that, for ε>0 small, there is a unique solution that is close to the singular orbit.

For our case, the singular orbit consists of nine pieces: two boundary layers $\Gamma_{l}^{0}$ and $\Gamma_{r}^{1}$; four internal layers $\Gamma_{l}^{a},\;\Gamma_{m}^{a},\;\Gamma_{m}^{b}$ and $\Gamma_{r}^{b}$; and three regular layers $\Lambda_{l},\;\Lambda_{m}$ and $\Lambda_{r}$ (see Figure 1). More precisely, with $J=(J_{1},J_{2},J_{3})$,

1. The boundary layer $\Gamma_{l}^{0}$ connects the point $\left(V,u_{r}^{0},L_{1},L_{2},L_{3},J,0\right)\in B_{L}$ to the point $\left(\phi^{L},0,c_{1}^{L},c_{2}^{L},c_{3}^{L},J,0\right)\in \omega \left(N_{l}^{0}\right)\subset Z_{l};$
2. The regular layer $\Lambda_{1}$ connects the point $\left(\phi^{L},0,c_{1}^{L},c_{2}^{L},c_{3}^{L},J,0\right)\in \omega \left(N_{l}^{0}\right)\subset Z_{l}$ to the point $\left(\phi^{\left[a,l\right]},0,c_{1}^{\left[a,l\right]},c_{2}^{\left[a,l\right]},c_{3}^{\left[a,l\right]},J,a\right)\in \alpha \left(N_{l}^{a}\right)\subset Z_{l};$
3. The internal layer $\Gamma_{l}^{a}$ connects the point $\left(\phi^{\left[a,l\right]},0,c_{1}^{\left[a,l\right]},c_{2}^{\left[a,l\right]},c_{3}^{\left[a,l\right]},J,a)\in \alpha (N_{l}^{a}\right)\subset Z_{l}$ to the point $\left(\phi^{\left[a\right]},u_{l}\left(a\right),c_{1}^{\left[a\right]},c_{2}^{\left[a\right]},c_{3}^{\left[a\right]},J,a\right)\in B_{a};$
4. The internal layer $\Gamma_{m}^{a}$ connects the point $\left(\phi^{\left[a\right]},u_{l}\left(a\right),c_{1}^{\left[a\right]},c_{2}^{\left[a\right]},c_{3}^{\left[a\right]},J,a\right)\in B_{a}$ to the point $\left(\phi^{\left[a,m\right]},0,c_{1}^{\left[a,m\right]},c_{2}^{\left[a,m\right]},c_{3}^{\left[a,m\right]},J,a\right)\in \omega \left(N_{m}^{a}\right)\subset Z_{m};$
5. The regular layer $\Lambda_{m}$ connects the point $\left(\phi^{\left[a,m\right]},0,c_{1}^{\left[a,m\right]},c_{2}^{\left[a,m\right]},c_{3}^{\left[a,m\right]},J,a\right)\in \omega \left(N_{m}^{a}\right)\subset Z_{m}$ to the point $\left(\phi^{\left[b,m\right]},0,c_{1}^{\left[b,m\right]},c_{2}^{\left[b,m\right]},c_{3}^{\left[b,m\right]},J,b\right)\in \alpha \left(N_{m}^{b}\right)\subset Z_{m};$
6. The internal layer $\Gamma_{m}^{b}$ connects the point $\left(\phi^{\left[b,m\right]},0,c_{1}^{\left[b,m\right]},c_{2}^{\left[b,m\right]},c_{3}^{\left[b,m\right]},J,b\right)\in \alpha \left(N_{m}^{b}\right)\subset Z_{m}$ to the point $\left(\phi^{\left[b\right]},u_{m}\left(b\right),c_{1}^{\left[b\right]},c_{2}^{\left[b\right]},c_{3}^{\left[b\right]},J,b\right)\in B_{b};$
7. The internal layer $\Gamma_{r}^{b}$ connects the point $\left(\phi^{\left[b\right]},u_{m}\left(b\right),c_{1}^{\left[b\right]},c_{2}^{\left[b\right]},c_{3}^{\left[b\right]},J,b\right)\in B_{b}$ to the point $\left(\phi^{\left[b,r\right]},0,c_{1}^{\left[b,r\right]},c_{2}^{\left[b,r\right]},c_{3}^{\left[b,r\right]},J,b\right)\in \omega \left(N_{r}^{b}\right)\subset Z_{r};$
8. The regular layer $\Lambda_{r}$ connects the point $\left(\phi^{\left[b,r\right]},0,c_{1}^{\left[b,r\right]},c_{2}^{\left[b,r\right]},c_{3}^{\left[b,r\right]},J,b\right)\in \omega \left(N_{r}^{b}\right)\subset Z_{r}$ to the point $\left(\phi^{R},0,c_{1}^{R},c_{2}^{R},c_{3}^{R},J,1\right)\in \alpha \left(N_{r}^{1}\right)\subset Z_{r};$
9. The boundary layer $\Gamma_{r}^{1}$ connects the point $\left(\phi^{R},0,c_{1}^{R},c_{2}^{R},c_{3}^{R},J,1\right)\in \alpha \left(N_{r}^{1}\right)\subset Z_{r}$ to the point $\left(0,u_{l}^{1},R_{1},R_{2},R_{3},J,1\right)\in B_{R}.$

The following result can be established by the exchange lemma (see, for example, [48,50,51]) of the geometric singular perturbation theory (see also [7,10,11,12]).

**Theorem** **1.** *Let $\Gamma_{l}^{0}\cup \Lambda_{l}\cup \Gamma_{l}^{a}\cup \Gamma_{m}^{a}\cup \Lambda_{m}\cup \Gamma_{m}^{b}\cup \Gamma_{r}^{b}\cup \Lambda_{r}\cup \Gamma_{r}^{1}$ be the singular orbit of the connecting problem system (10) associated with $B_{L}$ and $B_{R}$ in system (12). There exists $\varepsilon_{0}>0$ small, so that if $0<\varepsilon <\varepsilon_{0},$ then the boundary value problem (8) and (9) has a unique solution near the singular orbit $\Gamma_{l}^{0}\cup \Lambda_{l}\cup \Gamma_{l}^{a}\cup \Gamma_{m}^{a}\cup \Lambda_{m}\cup \Gamma_{m}^{b}\cup \Gamma_{r}^{b}\cup \Lambda_{r}\cup \Gamma_{r}^{1}$.*

**Proof.**  Fix δ>0 small to be determined. Let

$$B_{L}\left(\delta \right)=\left\{\left(V,u,L_{1},L_{2},L_{3},J_{1},J_{2},J_{3},0\right)\in R^{9}:|u-u_{r}^{0}\left|<\delta ,\right|J_{i}-J_{i}^{l}|<\delta \right\},$$

which is a neighborhood of $\Gamma_{l}^{0}\cap B_{L}=\left\{(V,u_{l}^{0},L_{1},L_{2},L_{3},J_{1}^{l},J_{2}^{l},J_{3}^{l},0)\right\}$ in $B_{L}$.

For ε>0, let $M_{l}^{0}\left(\varepsilon \right)$ be the forward trace of $B_{L}\left(\delta \right)$ under the flow of system (10). We will show that $M_{l}^{0}\left(\varepsilon \right)$ intersects $B_{R}$ transversally near the point $\Gamma_{r}^{1}\cap B_{R}=\{(0,u_{r}^{1},R_{1},R_{2},$ $R_{3},J_{1}^{r},J_{2}^{r},J_{3}^{r},1)\}.$

The evolution of $M_{l}^{0}\left(\varepsilon \right)$ will undergo 3 stages one over each subinterval [0,a], [a,b] and [b,1]. In the first stage over [0,a], it will start close to the point $\left(V,u_{l}^{0},L_{1},L_{2},L_{3},J_{1}^{l},J_{2}^{l},J_{3}^{l},0\right)$, follow the singular layer $\Gamma_{l}^{0}$ toward the slow manifold $Z_{l}$, move along the regular layer $\Lambda_{l}$, and leave the vicinity of $Z_{l}$ along the singular layer $\Gamma_{l}^{a}$ toward the point $(\phi^{\left[a,l\right]},u_{l}\left(a\right),c_{1}^{\left[a,l\right]},$ $c_{2}^{\left[a,l\right]},c_{3}^{\left[a,l\right]},J_{1}^{l},J_{2}^{l},J_{3}^{l},a)$. It then continues the evolution over each subsequent subintervals in a similar fashion until it reaches the vicinity of the point $\left(0,u_{r}^{1},R_{1},R_{2},R_{3},J_{1}^{r},J_{2}^{r},J_{3}^{r},1\right)\in B_{R}$.

To track this evolution of $M_{l}^{0}\left(\varepsilon \right)$, we apply the exchange lemma successively (three times) along the stages in order described above. During the first stage, we track the evolution of $M_{l}^{0}\left(\varepsilon \right)$ along the singular orbit $\Gamma_{l}^{0}\cup \Lambda_{l}\cup \Gamma_{l}^{a}$. The Exchange Lemma ([48,50,51], etc.) indicates that, at the end of the first stage and near $\Gamma_{l}^{a}$, $M_{l}^{0}\left(\varepsilon \right)$ is $C^{1}O\left(\varepsilon \right)$-close to $W^{u}\left(\alpha \left(N_{l}^{a}\right)\cdot \left(-\rho ,\rho \right)\right)$ for some ρ>0 independent of ε, provided that the following conditions are satisfied:

(i) $M_{l}^{0}\left(0\right)\cap W^{s}\left(Z_{l}\right)$ is transversal along $\Gamma_{l}^{0}$, which is established in Proposition 2;
(ii) the vector field on $Z_{l}$ is not tangent to $\omega (N_{l}^{0})$ at $\left(\phi^{L},0,c_{1}^{L},c_{2}^{L},c_{3}^{L},J_{1}^{l},J_{2}^{l},J_{3}^{l},0\right)\in Z_{l}$, which follows from $\dot{\tau }=1$ in (21).

Let $\Sigma_{l}=W^{u}(\alpha \left(N_{l}^{a}\right)\cdot \left(-\rho ,\rho \right)\cap \left\{\tau =a\right\}.$ then, near $\Gamma_{l}^{a}$, $M_{l}^{0}\left(\varepsilon \right)$ is close to the forward trace of $\Sigma_{l}$ under the flow of system (10) with $Q=Q_{0}$. We can then apply the Exchange Lemma again to $M_{l}\left(\varepsilon \right)$ along $\Gamma_{m}^{a}\cup \Lambda_{m}\cup \Gamma_{m}^{b}$ 0ver [a,b]. At the end of this stage, one has that $M_{l}\left(\varepsilon \right)$ is $C^{1}O\left(\varepsilon \right)$-close to $W^{u}\left(\alpha \left(N_{m}^{b}\right)\cdot \left(-\rho ,\rho \right)\right).$

Let $\Sigma_{m}=W^{u}(\alpha \left(N_{m}^{b}\right)\cdot \left(-\rho ,\rho \right)\cap \left\{\tau =b\right\}.$ then, near $\Gamma_{m}^{b}$, $M_{l}^{0}\left(\varepsilon \right)$ is close to the forward trace of $\Sigma_{m}$ under the flow of system (10) with Q=0. We can then apply the Exchange Lemma again to $M_{l}\left(\varepsilon \right)$ along $\Gamma_{r}^{b}\cup \Lambda_{r}\cup \Gamma_{r}^{1}$ 0ver [b,1]. At the end of this stage, one has that $M_{l}\left(\varepsilon \right)$ is $C^{1}O\left(\varepsilon \right)$-close to $W^{u}\left(\alpha \left(N_{r}^{1}\right)\cdot \left(-\rho ,\rho \right)\right).$ Since the latter is transversal to $B_{R}$ near the point $\left(0,u_{l}^{1},R_{1},R_{2},R_{3},J_{1}^{r},J_{2}^{r},J_{3}^{r},1\right)$, $M_{l}^{0}\left(\varepsilon \right)$ intersects $B_{R}$ transversally near $\left(0,u_{l}^{1},R_{1},R_{2},R_{3},J_{1}^{r},J_{2}^{r},J_{3}^{r},1\right)$. Note that $\dim M_{l}^{0}\left(\varepsilon \right)=\dim B_{L}+1=5$ and $\dim B_{R}=4$. Therefore, $\dim\left(M_{l}^{0}\left(\varepsilon \right)\cap B_{R}\right)=\dim M_{l}^{0}\left(\varepsilon \right)+\dim B_{R}-9=0;$ that is, the intersection near $\left(0,u_{l}^{1},R_{1},R_{2},R_{3},J_{1}^{r},J_{2}^{r},J_{3}^{r},1\right)$ is a singleton. This completes the proof. □

### 2.2. Regular Perturbation Analysis: Expansions along Small $Q_{0}$

We expand all unknown quantities in the governing system (40) and (41) in $Q_{0}$ under the assumption that $|Q_{0}|$ is small, for example, for k=1,2,3, we write

(42) $$\begin{array}{c} \begin{array}{cc} \phi^{\left[a\right]}= & \phi_{0}^{\left[a\right]}+\phi_{1}^{\left[a\right]}Q_{0}+\phi_{2}^{\left[a\right]}Q_{0}^{2}+o\left(Q_{0}^{2}\right),\;\phi^{\left[b\right]}=\phi_{0}^{\left[b\right]}+\phi_{1}^{\left[b\right]}Q_{0}+\phi_{2}^{\left[b\right]}Q_{0}^{2}+o\left(Q_{0}^{2}\right),\\ c_{k}^{\left[a\right]}= & c_{k0}^{\left[a\right]}+c_{k1}^{\left[a\right]}Q_{0}+c_{k2}^{\left[a\right]}Q_{0}^{2}+o\left(Q_{0}^{2}\right),\;c_{k}^{\left[b\right]}=c_{k0}^{\left[b\right]}+c_{k1}^{\left[b\right]}Q_{0}+c_{k2}^{\left[b\right]}Q_{0}^{2}+o\left(Q_{0}^{2}\right),\\ y_{0}= & y_{00}+y_{01}Q_{0}+y_{02}Q_{0}^{2}+o\left(Q_{0}^{2}\right),\;J_{k}=J_{k0}+J_{k1}Q_{0}+J_{k2}Q_{0}^{2}+o\left(Q_{0}^{2}\right). \end{array} \end{array}$$

We will determine the coefficients of the zeroth order and first order terms for dominating effects on ionic flows from the permanent charge. We introduce

(43) $$\begin{array}{c} \begin{array}{cc} \alpha = & H(a)/H(1),\;\beta =H(b)/H(1), \end{array} \end{array}$$

and, corresponding to (16) and (19),

(44) $$\begin{array}{c} \begin{array}{cc} T_{0}^{m}=J_{10}+J_{20}+J_{30},\;C_{i}^{\left[a\right]}= & c_{1i}^{\left[a\right]}+c_{2i}^{\left[a\right]},\;C_{i}^{b}=c_{1i}^{\left[b\right]}+c_{2i}^{\left[b\right]},\;i=0,1. \end{array} \end{array}$$

Careful calculations (tedious but straightforward) give

**Proposition** **6.** *The zeroth order solution in $Q_{0}$ of (40) and (41) is given by*

$$\begin{array}{c} \begin{array}{cc} \phi_{0}^{\left[a,l\right]} & =\phi_{0}^{\left[a,m\right]}=\phi_{0}^{\left[a\right]}=\frac{\ln C_{0}^{\left[a\right]}-\ln R}{\ln L-\ln R}V,\;\phi_{0}^{\left[b,m\right]}=\phi_{0}^{\left[b,r\right]}=\phi_{0}^{\left[b\right]}=\frac{\ln C_{0}^{\left[b\right]}-\ln R}{\ln L-\ln R}V,\\ c_{k0}^{\left[a,l\right]} & =c_{k0}^{\left[a,m\right]}=c_{k0}^{\left[a\right]}=\frac{C_{0}^{\left[a\right]}\left(L_{k}-R_{k}e^{-zV}\right)+\left(LR_{k}-L_{k}R\right)e^{-z\phi_{0}^{\left[a\right]}}}{L-Re^{-zV}},\\ c_{k0}^{\left[b,m\right]} & =c_{k0}^{\left[b,r\right]}=c_{k0}^{\left[b\right]}=\frac{C_{0}^{\left[b\right]}\left(L_{k}-R_{k}e^{-zV}\right)+\left(LR_{k}-L_{k}R\right)e^{-z\phi_{0}^{\left[b\right]}}}{L-Re^{-zV}},\\ J_{k0} & =\frac{L-R}{H\left(1\right)\left(\ln L-\ln R\right)}\frac{\ln L-\ln R+zV}{L-Re^{-zV}}\left(L_{k}-R_{k}e^{-zV}\right),\\ J_{30} & =-\frac{z}{z_{3}}\frac{L-R}{H\left(1\right)\left(\ln L-\ln R\right)}\left(\ln L-\ln R+z_{3}V\right),\\ y_{00} & =\frac{H\left(1\right)\left(\ln B-\ln C_{0}^{\left[a\right]}\right)}{z\left(z-z_{3}\right)\left(R-L\right)},\;c_{30}^{\left[a\right]}=-\frac{z}{z_{3}}C_{0}^{\left[a\right]},\;c_{30}^{\left[b\right]}=-\frac{z}{z_{3}}C_{0}^{\left[b\right]}, \end{array} \end{array}$$

*where $C_{0}^{\left[a\right]}=L+\alpha \left(R-L\right)$ and $C_{0}^{\left[b\right]}=L+\beta \left(R-L\right)$.*

**Proposition** **7.** *The first order solution in $Q_{0}$ of (40) and (41) is given by, with k=1,2,*

$$\begin{array}{c} \begin{array}{cc} \; & c_{k1}^{\left[a\right]}=\frac{\left(L_{k}-R_{k}e^{-zV}\right)\left(C_{1}^{\left[a\right]}+z\phi_{1}^{\left[a\right]}C_{0}^{\left[a\right]}\right)}{L-Re^{-zV}}-z\phi_{1}^{\left[a\right]}c_{k0}^{\left[a\right]},\\ \; & c_{k1}^{\left[b\right]}=\frac{\left(L_{k}-R_{k}e^{-zV}\right)\left(C_{1}^{\left[b\right]}+z\phi_{1}^{\left[b\right]}C_{0}^{\left[b\right]}\right)}{L-Re^{-zV}}-z\phi_{1}^{\left[b\right]}c_{k0}^{\left[b\right]},\\ \; & \phi_{1}^{\left[a\right]}=\frac{\left(1+z\lambda \right)\left(1+z_{3}\lambda \right)\left(C_{0}^{\left[a\right]}-C_{0}^{\left[b\right]}\right)\left(\ln L-\ln C_{0}^{\left[a\right]}\right)}{z\left(z-z_{3}\right)C_{0}^{\left[a\right]}C_{0}^{\left[b\right]}\left(\ln L-\ln R\right)}+\frac{1+2zz_{3}\alpha \left(\phi_{0}^{\left[b\right]}-\phi_{0}^{\left[a\right]}\right)\lambda }{2z\left(z-z_{3}\right)C_{0}^{\left[a\right]}},\\ \; & \phi_{1}^{\left[b\right]}=\frac{\left(1+z\lambda \right)\left(1+z_{3}\lambda \right)\left(C_{0}^{\left[a\right]}-C_{0}^{\left[b\right]}\right)\left(\ln R-\ln C_{0}^{\left[b\right]}\right)}{z\left(z-z_{3}\right)C_{0}^{\left[a\right]}C_{0}^{\left[b\right]}\left(\ln L-\ln R\right)}+\frac{1+2zz_{3}\left(1-\beta \right)\left(\phi_{0}^{\left[b\right]}-\phi_{0}^{\left[a\right]}\right)\lambda }{2z\left(z-z_{3}\right)C_{0}^{\left[b\right]}},\\ \; & y_{01}=\frac{\left(C_{0}^{\left[a\right]}\left(\beta -1\right)-C_{0}^{\left[b\right]}\alpha \right)\left(\phi_{0}^{b}-\phi_{0}^{a}\right)}{z\left(z-z_{3}\right)C_{0}^{\left[a\right]}C_{0}^{\left[b\right]}T_{0}^{m}}+\frac{\left(\ln C_{0}^{\left[a\right]}-\ln C_{0}^{\left[b\right]}\right)\left(\phi_{0}^{b}-\phi_{0}^{a}\right)}{z\left(z-z_{3}\right)\left(R-L\right)T_{0}^{m}}\\ \; & \;+\frac{\left(C_{0}^{\left[b\right]}-C_{0}^{\left[a\right]}\right)\left(z_{3}\left(J_{10}+J_{20}\right)+zJ_{30}\right)}{z^{2}z_{3}\left(z-z_{3}\right)C_{0}^{\left[a\right]}C_{0}^{\left[b\right]}{\left(T_{0}^{m}\right)}^{2}},\\ \; & J_{k1}=\frac{L_{k}-R_{k}e^{-zV}}{L-Re^{-zV}}\frac{A\left(1+z\lambda \right)(z_{3}\left(1-B\right)\lambda +1)}{\left(z-z_{3}\right)H\left(1\right)},\;J_{31}=\frac{A\left(1+z_{3}\lambda \right)\left(z\left(1-B\right)\lambda +1\right)}{\left(z_{3}-z\right)H\left(1\right)}, \end{array} \end{array}$$

*where*

$$\begin{array}{c} \begin{array}{cc} \; & C_{1}^{\left[a\right]}=\frac{z_{3}\alpha \left(\phi_{0}^{\left[b\right]}-\phi_{0}^{\left[a\right]}\right)}{z-z_{3}}-\frac{1}{2(z-z_{3})},\;C_{1}^{\left[b\right]}=\frac{z_{3}\left(\beta -1\right)\left(\phi_{0}^{\left[b\right]}-\phi_{0}^{\left[a\right]}\right)}{z-z_{3}}-\frac{1}{2(z-z_{3})},\\ \; & \lambda =\frac{V}{\ln L-\ln R},\;A=\frac{\left(R-L\right)\left(C_{0}^{\left[a\right]}-C_{0}^{\left[b\right]}\right)}{C_{0}^{\left[a\right]}C_{0}^{\left[b\right]}\left(\ln L-\ln R\right)},\\ \; & B=\frac{\ln C_{0}^{\left[b\right]}-\ln C_{0}^{\left[a\right]}}{A}=\frac{C_{0}^{\left[a\right]}C_{0}^{\left[b\right]}\left(\ln L-\ln R\right)\left(\ln C_{0}^{\left[b\right]}-\ln C_{0}^{\left[a\right]}\right)}{\left(R-L\right)\left(C_{0}^{\left[a\right]}-C_{0}^{\left[b\right]}\right)}. \end{array} \end{array}$$

For convenience, we introduce $f_{0}\left(L,R\right)$ and $g_{0}\left(L,R;V\right)$, which are defined by

$$\begin{array}{c} f_{0}\left(L,R\right)=\frac{L-R}{\ln L-\ln R},\;f_{1}\left(L,R;V\right)=\frac{\ln L-\ln R+zV}{L-Re^{-zV}}. \end{array}$$

**Lemma** **5.** *One has*

(i) *if L≠R, then $f_{0}\left(L,R\right)>0$;*
(ii) *if $L\neq Re^{-zV}$, then $f_{1}\left(L,R;V\right)>0$.*

From Propositions 6 and 7, one has

**Corollary** **1.** *Assume the electroneutrality boundary conditions (15). For k=1,2, one has*

(45) $$\begin{array}{c} \begin{array}{cc} J_{k0} & =\frac{f_{0}\left(L,R\right)f_{1}\left(L,R;V\right)}{H(1)}\left(L_{k}-R_{k}e^{-zV}\right),\\ J_{30} & =-\frac{z}{z_{3}}\frac{f_{0}\left(L,R\right)}{H(1)}\left(\ln L-\ln R+z_{3}V\right),\\ J_{k1} & =f_{1}\left(L,R;V\right)\frac{A\left(z_{3}\left(1-B\right)V+\ln L-\ln R\right)}{\left(z-z_{3}\right)H\left(1\right){\left(\ln L-\ln R\right)}^{2}}\left(L_{k}-R_{k}e^{-zV}\right),\\ J_{31} & =\frac{A\left(z_{3}V+\ln L-\ln R\right)\left(z(1-B)V+\ln L-\ln R\right)}{\left(z_{3}-z\right)H\left(1\right){\left(\ln L-\ln R\right)}^{2}}, \end{array} \end{array}$$

*where $A=\frac{\left(\alpha -\beta \right)\left(L-R\right)f_{0}\left(L,R\right)}{\omega (\alpha )\omega (\beta )},\;B=\frac{\ln\omega (\beta )-\ln\omega (\alpha )}{A}$ with ω(x)=(1−x)L+xR.*

For convenience in our following analysis, we introduce a function γ(t) for t>0 with

(46) $$\begin{array}{c} \begin{array}{c} \gamma \left(t\right)=\frac{t\ln t-t+1}{(t-1)\ln t},\;\mathrm{for}\;t\neq 1,\;\mathrm{and}\;\gamma \left(1\right)=\frac{1}{2}. \end{array} \end{array}$$

For γ(t), one can easily established the following properties.

**Lemma** **6.** *For t>0, one has $0<\gamma \left(t\right)<1,\;\gamma^{\prime }\left(t\right)>0,\;\lim_{t\to 0}\gamma \left(t\right)=0$ and $\lim_{t\to \infty }\gamma \left(t\right)=1$.*

**Lemma** **7.** *Set t=L/R. A has the same sign with that of R−L, that is, if t>1, then A<0, and if t<1, then A>0.*

**Definition** **1.** *We define the critical potentials $V_{1}$, $V_{2}$ and $V_{5}$ by*

$$\begin{array}{c} \begin{array}{cc} \; & L-Re^{-zV_{1}}=0,\;z_{3}\left(1-B\right)V_{2}+\ln L-\ln R=0,\;\;L_{d}-R_{d}e^{-zV_{3}}=0, \end{array} \end{array}$$

*where $L_{d}R_{d}>0$. Furthermore,*

$$\begin{array}{c} \begin{array}{cc} \; & V_{1}=\frac{1}{z}\ln\frac{R}{L},\;V_{2}=\frac{1}{z_{3}\left(1-B\right)}\ln\frac{R}{L},\;V_{3}=\frac{1}{z}\ln\frac{R_{d}}{L_{d}}. \end{array} \end{array}$$

We would like to point out that for the discussion in Section 3.1, the sign of the term A(1−B) is critical. While the sign of *A* can be determined by R−L as stated in Lemma 7, we now establish the result, which characterizes the sign of 1−B.

**Lemma** **8.** *Assume t=L/R>1 and γ(t) be as in (46). Then B>0, and 1−B→0 as t→1. Furthermore,*

(i) *if γ(t)≤α, then $\frac{z}{z_{3}}<0<1-B$ and $V_{1}<0<V_{2}$;*
(ii) *if $\alpha <\gamma \left(t\right)<\alpha -\frac{z}{z_{3}\ln t}$, then, there exists a unique $\beta_{1}\in \left(\alpha ,1\right)$ such that*
  (ii1) *$\frac{z}{z_{3}}<1-B<0$ and $V_{2}<V_{1}<0$, for $\beta \in (\alpha ,\beta_{1})$;*
  (ii2) *$\frac{z}{z_{3}}<1-B=0$, for $\beta =\beta_{1}$;*
  (ii3) *$\frac{z}{z_{3}}<0<1-B$ and $V_{1}<0<V_{2}$, for $\beta \in (\beta_{1},1)$.*
(iii) if $\gamma \left(t\right)>\alpha -\frac{z}{z_{3}\ln t}$, then, there exists a unique $\beta_{1}^{*}\in \left(\alpha ,\beta_{1}\right)$ such that
  (iii1) $1-B<\frac{z}{z_{3}}<0$ and $V_{1}<V_{2}<0$, for $\beta \in (\alpha ,\beta_{1}^{*})$;
  (iii2) $1-B=\frac{z}{z_{3}}<0$ and $V_{2}=V_{1}<0$, for $\beta =\beta_{1}^{*}$;
  (iii3) $\frac{z}{z_{3}}<1-B<0$ and $V_{2}<V_{1}<0$, for $\beta \in (\beta_{1}^{*},\beta_{1})$;
  (iii4) *$\frac{z}{z_{3}}<1-B=0$, for $\beta =\beta_{1}$;*
  (iii5) *$\frac{z}{z_{3}}<0<1-B$ and $V_{1}<0<V_{2}$, for $\beta \in (\beta_{1},1)$.*

**Proof.**  The statement that B>0 follows from the fact that both *A* and lnω(β)−lnω(α) have the same sign with that of R−L.

Rewrite 1−B as

$$\begin{array}{c} \begin{array}{c} 1-B=\frac{g(\beta )}{\left(\beta -\alpha \right){\left(t-1\right)}^{2}}, \end{array} \end{array}$$

where $g\left(\beta \right)=\left(\left(1-\alpha \right)t+\alpha \right)\left(\left(1-\beta \right)t+\beta \right)\ln t\ln\frac{(1-\beta )t+\beta }{(1-\alpha )t+\alpha }+\left(\beta -\alpha \right){\left(t-1\right)}^{2}.$ It follows directly that $\lim_{t\to 1}\left(1-B\right)=0$. Careful computations yield

$$\begin{array}{c} \begin{array}{c} \frac{d(1-B)}{d\beta }=\frac{g_{1}\left(\beta \right)}{{\left(\beta -\alpha \right)}^{2}{\left(t-1\right)}^{2}}, \end{array} \end{array}$$

where $g_{1}\left(\beta \right)=-{\left(\left(1-\alpha \right)t+\alpha \right)}^{2}\ln t\ln\frac{(1-\beta )t+\beta }{(1-\alpha )t+\alpha }+\left(\beta -\alpha \right){\left(t-1\right)}^{2}\left((\alpha -\gamma (t))\ln t-1\right),$ and further

$$\begin{array}{c} \begin{array}{cc} \; & g_{1}^{\prime }\left(\beta \right)=-{\left(\left(1-\alpha \right)t+\alpha \right)}^{2}\frac{1-t}{(1-\beta )t+\beta }\ln t+{\left(t-1\right)}^{2}\left((\alpha -\gamma (t))\ln t-1\right),\\ \; & g_{1}^{\prime \prime }\left(\beta \right)={\left(\frac{(1-\alpha )t+\alpha }{(1-\beta )t+\beta }\right)}^{2}{\left(1-t\right)}^{2}\ln t. \end{array} \end{array}$$

One has $g_{1}^{\prime \prime }\left(\beta \right)>0$ for all t>1, that is, $g_{1}\left(\beta \right)$ is concave upward for t>1. Furthermore, one has $\lim_{\beta \to \alpha }g_{1}\left(\beta \right)=0$ and $\lim_{\beta \to \alpha }g_{1}^{\prime }\left(\beta \right)=0$, which indicate that $g_{1}\left(\beta \right)>0$ for β>α. Note that $\lim_{\beta \to \alpha }\frac{d(1-B)}{d\beta }=\ln t/2>0$ for t>1. Then, $\frac{d(1-B)}{d\beta }>0$ for β>α, and 1−B is strictly increasing on (α,+∞). Note also that $\lim_{\beta \to \alpha }\left(1-B\right)=\left(\alpha -\gamma \left(t\right)\right)\ln t$. One has, for t>1,

- if α≥γ(t), then, $\frac{z}{z_{3}}<0<1-B$, which implies $V_{1}<0<V_{2}$; this completes the proof of statement (i).
- For the case with α<γ(t), we first claim that there exists a unique $\beta_{1}\in \left(\alpha ,1\right)$ such that 1−B=0 for $\beta =\beta_{1}$. In fact, based on the facts that $\lim_{\beta \to \alpha }\left(1-B\right)=\left(\alpha -\gamma \left(t\right)\right)\ln t<0$ and 1−B is strictly increasing on (α,+∞), it suffices to show that 1−B>0 for β=1, which follows from g(1)>0. For convenience, for t>1, we set
  $$\begin{array}{c} \begin{array}{c} g_{2}\left(\alpha \right):=g\left(1\right)=-\left(\left(1-\alpha \right)t+\alpha \right)\ln t\ln\left((1-\alpha )t+\alpha \right)+\left(1-\alpha \right){\left(t-1\right)}^{2}. \end{array} \end{array}$$
  Direct calculations give $g_{2}^{\prime \prime }\left(\alpha \right)=-\frac{{\left(1-t\right)}^{2}\ln t}{(1-\alpha )t+\alpha }<0$ for all t>1, and hence, $g_{2}\left(\alpha \right)$ is concave downward for t>1. Further, g(1)>0 is implied by $g_{2}\left(0\right)\geq 0$, since $g_{2}\left(1\right)=0$. To prove $g_{2}\left(0\right)\geq 0$, set
  $$\begin{array}{c} \begin{array}{c} g_{3}\left(t\right):=g_{2}\left(0\right)=-t{\left(\ln t\right)}^{2}+{\left(t-1\right)}^{2}. \end{array} \end{array}$$
  It is easy to check that $g_{3}^{\prime }\left(t\right)=-{\left(\ln t\right)}^{2}-2\ln t+2\left(t-1\right)$ and $g_{3}^{\prime \prime }\left(t\right)=\frac{2}{t}\left(t-1-\ln t\right)>0$. Further one arrive at the conclusion that $g_{3}\left(t\right)=g_{2}\left(0\right)>0$ based on the facts that $g_{3}\left(1\right)=g_{3}^{\prime }\left(1\right)=0$ and $g_{3}^{\prime \prime }\left(t\right)>0$ for t>1.
  - If $\alpha <\gamma \left(t\right)<\alpha -\frac{z}{z_{3}\ln t}$, then one can easily obtain $\frac{z}{z_{3}}<1-B$ for all β>α, and more specifically, $\frac{z}{z_{3}}<1-B<0$, which implies $V_{2}<V_{1}<0$, for $\beta \in (\alpha ,\beta_{1})$, $\frac{z}{z_{3}}<1-B=0$, for $\beta =\beta_{1}$, $\frac{z}{z_{3}}<0<1-B$, which indicates $V_{1}<0<V_{2}$, for $\beta \in (\beta_{1},1)$; this completes the proof of statement (ii).
  - if $\gamma \left(t\right)>\alpha -\frac{z}{z_{3}\ln t}$, then, the straight line $w=\frac{z}{z_{3}}$ and w=1−B have the unique intersection $\left(\beta_{1}^{*},w\left(\beta_{1}^{*}\right)\right)$, which means that there exists a unique $\beta_{1}^{*}\in \left(\alpha ,\beta_{1}\right)$ such that $1-B<\frac{z}{z_{3}}<0$, which suggests $V_{1}<V_{2}<0$, for $\beta \in (\alpha ,\beta_{1}^{*})$, $1-B=\frac{z}{z_{3}}<0$ and further $V_{2}=V_{1}<0$, for $\beta =\beta_{1}^{*}$, $\frac{z}{z_{3}}<1-B<0$, which yields $V_{2}<V_{1}<0$, for $\beta \in (\beta_{1}^{*},\beta_{1})$, $\frac{z}{z_{3}}<1-B=0$, for $\beta =\beta_{1}$, $\frac{z}{z_{3}}<0<1-B$, which hints $V_{1}<0<V_{2}$, for $\beta \in (\beta_{1},1)$; this completes the proof of statement (iii).

 □

## 3. Results

### 3.1. Competitions between Cations

We now consider the competition between two positively charged ion species due to the small permanent charges, which further depends on the nonlinear interplays with other system parameters, such as channel geometry (α,β), diffusion coefficients $\left(D_{1},D_{2}\right)$ and boundary concentrations $\left(L_{1},L_{2},R_{1},R_{2}\right)$. The study is closely related to the selectivity phenomena of ion channels.

For convenience, we define $J_{1,2}$ as

(47) $$\begin{array}{c} \begin{array}{cc} J_{1,2}= & D_{1}J_{1}-D_{2}J_{2}=J_{1,2}^{0}+J_{1,2}^{1}Q_{0}+O\left(Q_{0}^{2}\right), \end{array} \end{array}$$

where $J_{1,2}^{0}=D_{1}J_{10}-D_{2}J_{20}$ and $J_{1,2}^{1}=D_{1}J_{11}-D_{2}J_{21}$ are given by

(48) $$\begin{array}{c} \begin{array}{cc} J_{1,2}^{0}= & \frac{f_{0}\left(L,R\right)}{H(1)}\frac{\ln L-\ln R+zV}{L-Re^{-zV}}\left(L_{d}-R_{d}e^{-zV}\right),\\ J_{1,2}^{1}= & \frac{Azz_{3}\left(1-B\right)}{\left(z-z_{3}\right)H\left(1\right){\left(\ln L-\ln R\right)}^{2}}\frac{\left(V-V_{1}\right)\left(V-V_{2}\right)}{L-Re^{-zV}}\left(L_{d}-R_{d}e^{-zV}\right), \end{array} \end{array}$$

where $L_{d}$ and $R_{d}$ is defined in (16). In particular, for B=1, one has

(49) $$\begin{array}{cc} J_{1,2}^{1}= & \frac{\ln L-\ln R+zV}{L-Re^{-zV}}\frac{A\left(L_{d}-R_{d}e^{-zV}\right)}{\left(z-z_{3}\right)H\left(1\right)\left(\ln L-\ln R\right)}. \end{array}$$

$J_{1,2}^{1}$ is the leading term that contains the effect from small permanent charges, and will be our main focus. The study on $J_{1,2}^{1}$ could provide important insights and better understanding of the selectivity of cations, and meanwhile demonstrates the critical role played by the small permanent charge in the selectivity of ion channels. The two expressions for $J_{1,2}^{1}\left(V\right)$ will be alternatively chosen for convenience in our following discussion.

In terms of the interplay between the diffusion coefficients and boundary concentrations, there are six cases to discuss, more precisely, one has

$$\begin{array}{cc} \; & \left(i\right)\;\frac{D_{1}}{D_{2}}=\frac{L_{2}}{L_{1}};\;\left(ii\right)\;\frac{D_{1}}{D_{2}}=\frac{R_{2}}{R_{1}};\;\left(iii\right)\;\frac{D_{1}}{D_{2}}<\min\left\{\frac{L_{2}}{L_{1}},\frac{R_{2}}{R_{1}}\right\};\;\left(iv\right)\;\frac{D_{1}}{D_{2}}>\max\left\{\frac{L_{2}}{L_{1}},\frac{R_{2}}{R_{1}}\right\};\\ \; & \left(v\right)\;\frac{R_{2}}{R_{1}}<\frac{D_{1}}{D_{2}}<\frac{L_{2}}{L_{1}};\;\left(vi\right)\;\frac{L_{2}}{L_{1}}<\frac{D_{1}}{D_{2}}<\frac{R_{2}}{R_{1}}. \end{array}$$

In this work, we will just focus on case (i), (iii) and (v), since the discussions for case (ii), (iv) and (vi) are very similar. Interested readers can work on them following our arguments.

In our following discussion, we examine $J_{1,2}^{1}\left(V\right)$ from two directions for each case: the sign of $J_{1,2}^{1}\left(V\right)$ and the monotonicity of $J_{1,2}^{1}\left(V\right)$ in the electric potential *V*.

#### 3.1.1. Case Study with $\frac{D_{1}}{D_{2}}=\frac{L_{2}}{L_{1}}$

In this section, we study $J_{1,2}\left(V\right)$ under the condition $\frac{D_{1}}{D_{2}}=\frac{L_{2}}{L_{1}},$ which is equivalent to $L_{d}=0.$

We first consider a special case.

**Theorem** **2.** *Suppose B=1 and $\frac{D_{1}}{D_{2}}=\frac{L_{2}}{L_{1}}$. One has $J_{1,2}^{1}\left(V\right)<0$ (resp. $J_{1,2}^{1}\left(V\right)>0$) if $\frac{D_{1}}{D_{2}}<\frac{R_{2}}{R_{1}}$ (resp. $\frac{D_{1}}{D_{2}}>\frac{R_{2}}{R_{1}}$), that is, the (small) positive $Q_{0}$ reduces (resp. enhances) $J_{1,2}$. Furthermore, $J_{1,2}^{1}\left(V\right)$ is increasing (resp. decreasing) in the potential V if $\frac{D_{1}}{D_{2}}<\frac{R_{2}}{R_{1}}$ (resp. $\frac{D_{1}}{D_{2}}>\frac{R_{2}}{R_{1}}$).*

**Proof.**  With $\frac{D_{1}}{D_{2}}=\frac{L_{2}}{L_{1}}$ and B=1, directly from (49), one has

$$J_{1,2}^{1}=-\frac{\ln L-\ln R+zV}{L-Re^{-zV}}\frac{AR_{d}e^{-zV}}{\left(z-z_{3}\right)H\left(1\right)\left(\ln L-\ln R\right)}.$$

From Lemmas 5 and 7, together with $z-z_{3}>0$ and H(1)>0, one has $J_{1,2}^{1}<0$ (resp. $J_{1,2}^{1}>0$) if $\frac{D_{1}}{D_{2}}<\frac{R_{2}}{R_{1}}$ (resp. $\frac{D_{1}}{D_{2}}>\frac{R_{2}}{R_{1}}$). To see the monotonicity of $J_{1,2}^{1}\left(V\right)$ in the potential *V*, we calculate

$$\frac{dJ_{1,2}^{1}\left(V\right)}{dV}=-\frac{zR_{d}Ae^{-zV}}{\left(z_{1}-z_{3}\right)H\left(1\right)\left(\ln L-\ln R\right)}\frac{h_{0}\left(V\right)}{{\left(L-Re^{-zV}\right)}^{2}},$$

where $h_{0}\left(V\right)=L-Re^{-zV}-L\left(\ln L-\ln R+zV\right).$ Direct calculation shows that $h_{0}\left(V\right)$ is concave downward for all *V* and attains its global maximum 0 at $V_{1}=\frac{1}{z}\ln\frac{R}{L}$. Note that $\lim_{V\to V_{1}}\frac{h_{0}\left(V\right)}{{\left(L-re^{-zV}\right)}^{2}}=-\frac{1}{2L}.$ One then has $\frac{dJ_{1,2}^{1}\left(V\right)}{dV}<0$ (resp. $\frac{dJ_{1,2}^{1}\left(V\right)}{dV}>0$) if $R_{d}>0$, that is, $\frac{D_{1}}{D_{2}}>\frac{R_{2}}{R_{1}}$ (resp. $R_{d}<0$, that is, $\frac{D_{1}}{D_{2}}<\frac{R_{2}}{R_{1}}$). This completes our proof. □

We next consider the more general case with B≠1.

**Theorem** **3.** *Suppose that B≠1. If $\frac{D_{1}}{D_{2}}=\frac{L_{2}}{L_{1}}$, one has*

(i) *if $A\left(1-B\right)R_{d}>0$, then, $J_{1,2}^{1}<0$ (resp. $J_{1,2}^{1}>0$), for $V<V_{2}$ (resp. $V>V_{2}$), that is, the (small) positive $Q_{0}$ reduces (resp. enhances) $J_{1,2}$;*
(ii) *if $A\left(1-B\right)R_{d}<0$, then, $J_{1,2}^{1}>0$ (resp. $J_{1,2}^{1}<0$), for $V<V_{2}$ (resp. $V>V_{2}$), that is, the (small) positive $Q_{0}$ enhances (resp. reduces) $J_{1,2}$.*

**Proof.**  The statements follow directly from (49) and Lemma 5. □

**Theorem** **4.** *Suppose that B≠1 and $\frac{D_{1}}{D_{2}}=\frac{L_{2}}{L_{1}}$. One has*

(i) *if $A\left(1-B\right)R_{d}>0$, then, there exists a critical potential $V_{c}^{21}$ with $V_{c}^{21}>V_{2}$ such that $J_{1,2}^{1}$ increases on $\left(-\infty ,V_{c}^{21}\right)$ and decreases on $\left(V_{c}^{21},\infty \right)$.*
(ii) *if $A\left(1-B\right)R_{d}<0$, then, there exists a critical potential $V_{c}^{22}$ with $V_{c}^{22}>V_{2}$ such that $J_{1,2}^{1}$ decreases on $\left(-\infty ,V_{c}^{22}\right)$ and increases on $\left(V_{c}^{22},\infty \right)$.*

**Proof.**  We will just provide a detailed proof for the first statement, and the second one can be discussed similarly. With $\frac{D_{1}}{D_{2}}=\frac{L_{2}}{L_{1}}$, one has

$$\begin{array}{c} \begin{array}{cc} J_{1,2}^{1}\left(V\right) & =\frac{-zz_{3}A\left(1-B\right)R_{d}}{\left(z-z_{3}\right)H\left(1\right){\left(\ln L-\ln R\right)}^{2}}\frac{\left(V-V_{1}\right)\left(V-V_{2}\right)}{Le^{zV}-R}. \end{array} \end{array}$$

It follows that

$$\begin{array}{c} \begin{array}{c} \frac{dJ_{1,2}^{1}}{dV}=\frac{-zz_{3}A\left(1-B\right)R_{d}}{\left(z-z_{3}\right)H\left(1\right){\left(\ln L-\ln R\right)}^{2}}\frac{e^{zV}}{{\left(Le^{zV}-R\right)}^{2}}f_{d}\left(V\right), \end{array} \end{array}$$

where $f_{d}\left(V\right)=\left(2V-V_{1}-V_{2}\right)\left(L-Re^{-zV}\right)-zL\left(V-V_{1}\right)\left(V-V_{2}\right),$ and further

$$\begin{array}{c} \begin{array}{c} f_{d}^{\prime }\left(V\right)=\frac{df_{d}}{dV}=\left(L-Re^{-zV}\right)\left(2-z\left(2V-V_{1}-V_{2}\right)\right). \end{array} \end{array}$$

It is easy to see that $f_{d}^{\prime }\left(V\right)$ has two zeros given by $V_{1}$ and $V_{d}=\frac{1}{2}\left(V_{1}+V_{2}+\frac{2}{z}\right)$. Furthermore, one can easily verify that $V_{1}<V_{d}$ if $2-z\left(V_{1}-V_{2}\right)>0$; $V_{1}=V_{d}$ if $2-z\left(V_{1}-V_{2}\right)=0$; and $V_{1}>V_{d}$ if $2-z\left(V_{1}-V_{2}\right)<0$.

If $V_{1}<V_{d2}$, then, $f_{d}\left(V\right)$ decreases on $\left(-\infty ,V_{1}\right)$, increases on $\left(V_{1},V_{d}\right)$, and decreases on $\left(V_{d},\infty \right)$. Note that $f_{d}\left(V_{1}\right)=0$, which is a local minimum of $f_{d}\left(V\right)$, $\lim_{V\to -\infty }f_{d}\left(V\right)=\infty$ and $\lim_{V\to \infty }f_{d}\left(V\right)=-\infty$. The function $f_{d}\left(V\right)$ has a second zero, denoted by $V_{c}^{21}$, with $V_{c}^{21}>V_{1}$. Furthermore, for $A\left(1-B\right)R_{d}>0$, we have

$$\lim_{V\to V_{1}}\frac{dJ_{1,2}^{1}\left(V\right)}{dV}=\frac{-z_{3}A\left(1-B\right)R_{d}R\left(2-z\left(V_{1}-V_{2}\right)\right)}{2\left(z-z_{3}\right)H\left(1\right){\left(\ln L-\ln R\right)}^{2}L^{2}e^{2zV_{1}}}>0,$$

since $2-z\left(V_{1}-V_{2}\right)>2-z\left(2V_{d}-V_{1}-V_{2}\right)=0$, which can be obtained from the fact that $2-z\left(2V-V_{1}-V_{2}\right)$ decreases on $\left(-\infty ,V_{d}\right)$ and $V_{1}<V_{d}$. Therefore, $\frac{dJ_{1,2}^{1}\left(V\right)}{dV}>0$ if $V<V_{c}^{21}$, and $\frac{dJ_{1,2}^{1}\left(V\right)}{dV}<0$ if $V>V_{c}^{21}$. This holds for the case with $V_{1}\geq V_{d}$ by a similar argument. Note that $\lim_{V\to \infty }J_{1,2}^{1}\left(V\right)=0$ and $\lim_{V\to -\infty }J_{1,2}^{1}\left(V\right)=-\infty$. Statement (i) then follows. □

#### 3.1.2. Case Study with $\frac{D_{1}}{D_{2}}<\min\left\{\frac{L_{2}}{L_{1}},\frac{R_{2}}{R_{1}}\right\}$

We study the term $J_{1,2}^{1}\left(V\right)$, which provides information of the preference of the ion channel over different cation under the condition $\frac{D_{1}}{D_{2}}<\min\left\{\frac{L_{2}}{L_{1}},\frac{R_{2}}{R_{1}}\right\}$.

We first characterize the sign of A(1−B) under the further restriction $\frac{D_{1}}{D_{2}}<\min\left\{\frac{L_{2}}{L_{1}},\frac{R_{2}}{R_{1}}\right\}$, and the order of the critical potentials $V_{2}$ and $V_{3}$.

**Corollary** **2.** Let $t=\frac{L}{R}>1$ and γ(t) be as in (46). Suppose $\frac{D_{1}}{D_{2}}<\min\left\{\frac{L_{2}}{L_{1}},\frac{R_{2}}{R_{1}}\right\}$. One has A(1−B)>0 and $V_{2}>V_{3}$ if $t<\frac{L_{d}}{R_{d}}$, $\gamma \left(t\right)>\alpha -\frac{z}{z_{3}\ln t}$ and $\beta \in (\alpha ,\beta_{1}^{*}]$.

**Corollary** **3.** *Let $t=\frac{L}{R}>1$ and γ(t) be as in (46). Suppose $\frac{D_{1}}{D_{2}}<\min\left\{\frac{L_{2}}{L_{1}},\frac{R_{2}}{R_{1}}\right\}$. One has A(1−B)<0 and $V_{2}>V_{3}$ under one of the following conditions*

(i) *$\frac{L_{d}}{R_{d}}>1$ and γ(t)≤α;*
(ii) *$\frac{L_{d}}{R_{d}}>1$ and $\alpha <\gamma \left(t\right)<\alpha -\frac{z}{z_{3}\ln t}$ and $\beta \in (\beta_{1},1)$;*
(iii) *$\frac{L_{d}}{R_{d}}>1$ and $\gamma \left(t\right)>\alpha -\frac{z}{z_{3}\ln t}$ and $\beta \in (\beta_{1},1)$.*

**Corollary** **4.** *Let $t=\frac{L}{R}>1$ and γ(t) be as in (46). Suppose $\frac{D_{1}}{D_{2}}<\min\left\{\frac{L_{2}}{L_{1}},\frac{R_{2}}{R_{1}}\right\}$. One has A(1−B)>0 and $V_{2}<V_{3}$ under one of the following conditions*

(i) *$t>1>\frac{L_{d}}{R_{d}}$, $\alpha <\gamma \left(t\right)<\alpha -\frac{z}{z_{3}\ln t}$ and $\beta \in (\alpha ,\beta_{1})$;*
(ii) *$t>\frac{L_{d}}{R_{d}}>1$, $\alpha <\gamma \left(t\right)<\alpha -\frac{z}{z_{3}\ln t}$ and $\beta \in (\alpha ,\beta_{1})$;*
(iii) *$t>1>\frac{L_{d}}{R_{d}}$, $\gamma \left(t\right)>\alpha -\frac{z}{z_{3}\ln t}$ and $\beta \in (\alpha ,\beta_{1})$;*
(iv) $t>\frac{L_{d}}{R_{d}}>1$, $\gamma \left(t\right)>\alpha -\frac{z}{z_{3}\ln t}$ and $\beta \in (\beta_{1}^{*},\beta_{1})$.

We comment that the proofs of Corollaries 2–4 can be directly discussed based on Lemmas 7 and 8. We skip the details here.

**Theorem** **5.** *Suppose that B=1 and $\frac{D_{1}}{D_{2}}<\min\left\{\frac{L_{2}}{L_{1}},\frac{R_{2}}{R_{1}}\right\}$. One has, $J_{1,2}^{1}\left(V\right)<0$ (resp. $J_{1,2}^{1}\left(V\right)>0$) if $V<V_{3}$ (resp. $V>V_{3}$), that is, the (small) positive $Q_{0}$ reduces (resp. enhances) $J_{1,2}\left(V\right)$. Furthermore, $J_{1,2}^{1}\left(V\right)$ increases in the potential V.*

**Theorem** **6.** *Suppose that B≠1 and $\frac{D_{1}}{D_{2}}<\min\left\{\frac{L_{2}}{L_{1}},\frac{R_{2}}{R_{1}}\right\}$. Then,*

(i) *For A(1−B)>0 and $V_{2}>V_{3},$ one has, $J_{1,2}^{1}\left(V\right)>0$ (resp. $J_{1,2}^{1}\left(V\right)<0$) if $V<V_{3}$ or $V>V_{2}$ (resp. $V_{3}<V<V_{2}$), that is, the (small) positive $Q_{0}$ enhances (resp. reduces) $J_{1,2}\left(V\right)$;*
(ii) *For A(1−B)<0 and $V_{2}>V_{3}$, one has, $J_{1,2}^{1}\left(V\right)<0$ (resp. $J_{1,2}^{1}\left(V\right)>0$) if $V<V_{3}$ or $V>V_{2}$ (resp. $V_{3}<V<V_{2}$), that is, the (small) positive $Q_{0}$ reduces (resp. enhances) $J_{1,2}\left(V\right)$;*
(iii) *For A(1−B)>0 and $V_{2}<V_{3}$, one has, $J_{1,2}^{1}\left(V\right)>0$ (resp. $J_{1,2}^{1}\left(V\right)<0$) if $V<V_{2}$ or $V>V_{3}$ (resp. $V_{2}<V<V_{3}$), that is, the (small) positive $Q_{0}$ enhances (resp. reduces) $J_{1,2}\left(V\right)$.*

**Theorem** **7.** *Suppose that B≠1 and $\frac{D_{1}}{D_{2}}<\min\left\{\frac{L_{2}}{L_{1}},\frac{R_{2}}{R_{1}}\right\}$. One has*

(i) *If A(1−B)>0, then, there exists a critical $V_{c}^{1}$ between $V_{2}$ and $V_{3}$ such that $J_{1,2}^{1}\left(V\right)$ decreases on $\left(-\infty ,V_{c}^{1}\right)$ and increases on $\left(V_{c}^{1},+\infty \right)$;*
(ii) *If A(1−B)<0, then, there exists a critical $V_{c}^{2}$ between $V_{2}$ and $V_{3}$ such that $J_{1,2}^{1}\left(V\right)$ increases on $\left(-\infty ,V_{c}^{2}\right)$ and decreases on $\left(V_{c}^{2},+\infty \right)$.*

#### 3.1.3. Case Study with $\frac{R_{2}}{R_{1}}<\frac{D_{1}}{D_{2}}<\frac{L_{2}}{L_{1}}$

In this section, we study the term $J_{1,2}^{1}\left(V\right)$ for the case with $\frac{R_{2}}{R_{1}}<\frac{D_{1}}{D_{2}}<\frac{L_{2}}{L_{1}}$, which is equivalent to $L_{d}<0$ and $R_{d}>0$. By similar arguments as those in Section 3.1.1 and Section 3.1.2, we obtain the following results.

**Theorem** **8.** *Suppose that B=1 and $\frac{R_{2}}{R_{1}}<\frac{D_{1}}{D_{2}}<\frac{L_{2}}{L_{1}}$. Then, $J_{1,2}^{1}\left(V\right)>0$, that is, the (small) positive $Q_{0}$ enhances $J_{1,2}\left(V\right)$.*

**Theorem** **9.** *Suppose that B≠1 and $\frac{R_{2}}{R_{1}}<\frac{D_{1}}{D_{2}}<\frac{L_{2}}{L_{1}}$. One has*

(i) *For A(1−B)>0,*
  (i1) *$J_{1,2}^{1}\left(V\right)<0$ (resp. $J_{1,2}^{1}\left(V\right)>0$) if $V<V_{2}$ (resp. $V>V_{2}$), that is, the (small) positive $Q_{0}$ reduces (resp. enhances) $J_{1,2}\left(V\right)$;*
  (i2) *$J_{1,2}^{1}\left(V\right)$ either always increases or there exist two critical $V_{c}^{3}$ and $V_{c}^{4}$ with $V_{c}^{3}<V_{c}^{4}$ such that $J_{1,2}^{1}\left(V\right)$ increases on $\left(-\infty ,V_{c}^{3}\right)$, decreases on $\left(V_{c}^{3},V_{c}^{4}\right)$ and increases on $\left(V_{c}^{4},\infty \right)$;*
(ii) *For A(1−B)<0,*
  (ii1) *$J_{1,2}^{1}\left(V\right)>0$ (resp. $J_{1,2}^{1}\left(V\right)<0$) if $V<V_{2}$ (resp. $V>V_{2}$), that is, the (small) positive $Q_{0}$ enhances (resp. reduces) $J_{1,2}\left(V\right)$.*
  (ii2) *$J_{1,2}^{1}\left(V\right)$ either always decreases or there exist two critical $V_{c}^{5}$ and $V_{c}^{6}$ with $V_{c}^{5}<V_{c}^{6}$ such that $J_{1,2}^{1}\left(V\right)$ decreases on $\left(-\infty ,V_{c}^{5}\right)$, increases on $\left(V_{c}^{5},V_{c}^{6}\right)$ and decreases on $\left(V_{c}^{6},\infty \right)$.*

#### 3.1.4. Studies on the Magnitude of ${\;J}_{1,2}\left(V\right)$

For convenience in the argument, we let $S_{1}$ represent the first cation corresponding to the flux $J_{1}$ and $S_{2}$ be the cation corresponding to the flux $J_{2}$.

Recall that, with $Q_{0}>0$ small, one has $J_{1,2}\left(V,Q_{0}\right)=J_{1,2}^{0}\left(V\right)+Q_{0}J_{1,2}^{1}\left(V\right)+o\left(Q_{0}\right)$. Further depending on the interaction among $\frac{D_{1}}{D_{2}},\frac{R_{2}}{R_{1}}$ and $\frac{L_{2}}{L_{1}}$, in Section 3.1.1, Section 3.1.2 and Section 3.1.3, we analyze the leading term $J_{1,2}^{1}\left(V\right)$ that contains the effects from small permanent charges, in particular,

(i) the sign of $J_{1,2}^{1}\left(V\right)$, which characterizes the small positive permanent charge effects on the competition between two cations. To be specific, if $J_{1,2}^{1}\left(V\right)>0$ (resp. $J_{1,2}^{1}\left(V\right)<0$), then, the small positive permanent charge enhances (resp. reduces) $J_{1,2}\left(V;Q_{0}\right)$, and in either way, it affects the preference of the ion channel over different cation, which is closely related to the selectivity phenomena of the ion channel.
(ii) the monotonicity of $J_{1,2}^{1}\left(V\right)$ in the electric potential *V*, which further reduces or strengthens the preference by adjusting/controlling the boundary membrane potential. Taking the case $\frac{dJ_{1,2}^{1}\left(V\right)}{dV}>0$ for example, if $J_{1,2}^{1}\left(V\right)>0$, then, one can increase the boundary electric potential to further strengthen the individual flux $J_{1}\left(V\right)$, which indicates that more cation $S_{1}$ will go through the ion channel; while if $J_{1,2}^{1}\left(V\right)<0$, one then need to decrease the boundary electric potential for more cation $S_{1}$ to go through the ion channels.

On the other hand, $J_{1,2}^{1}\left(V\right)>0$ (resp. $J_{1,2}^{1}\left(V\right)<0$), indicates $J_{1,2}\left(V,Q_{0}\right)>J_{1,2}\left(V;0\right)$ (resp. $J_{1,2}\left(V,Q_{0}\right)<J_{1,2}\left(V;0\right)$), but it does not provide any information on the relation of $|J_{1,2}\left(V;Q_{0}\right)|$ and $|J_{1,2}\left(V,0\right)|$, which contains complementary information for the competition, and further depends on the sign of $J_{1,2}^{0}\left(V\right)$.

Recall form Lemma 7 that A<0 if L>R, together with (48) and (49), one has

**Theorem** **10.** *Suppose that B=1. One has $J_{1,2}^{0}\left(V\right)J_{1,2}^{1}\left(V\right)<0$, that is, the (small) positive $Q_{0}$ reduces $|J_{1,2}\left(V\right)|$.*

**Theorem** **11.** *Suppose that B≠1. For t=L/R>1, one has A<0 and*

(i) *if either α≥γ(t), or α<γ(t) and $\beta \in (\beta_{1},1)$, then 1−B>0 and*
  (i1) *for $V>V_{2}$, $J_{1,2}^{0}\left(V\right)J_{1,2}^{1}\left(V\right)>0$, equivalently, (small) positive $Q_{0}$ enhances $|J_{1,2}\left(V\right)|$;*
  (i2) *for $V<V_{2}$, $J_{1,2}^{0}\left(V\right)J_{1,2}^{1}\left(V\right)<0$, equivalently, (small) positive $Q_{0}$ reduces $|J_{1,2}\left(V\right)|$;*
(ii) *if α<γ(t) and $\beta \in (\alpha ,\beta_{1})$, then 1−B<0 and*
  (ii1) *for $V<V_{2}$, $J_{1,2}^{0}\left(V\right)J_{1,2}^{1}\left(V\right)>0$, equivalently, (small) positive $Q_{0}$ enhances $|J_{1,2}\left(V\right)|$;*
  (ii2) *for $V>V_{2}$, $J_{1,2}^{0}\left(V\right)J_{1,2}^{1}\left(V\right)<0$, equivalently, (small) positive $Q_{0}$ reduces $|J_{1,2}\left(V\right)|$;*

**Proof.**  From (48), one note that the sign of $J_{1,2}^{0}\left(V\right)J_{1,2}^{1}\left(V\right)$ is determined by the sign of $Az_{3}\left(1-B\right)\left(V-V_{2}\right)$. Together with Lemmas 7 and 8, one can easily established the result. □

We point out that the studies provides further information of the preference of the ion channel over distinct cations. To be specific, we take $J_{1,2}^{0}\left(V\right)J_{1,2}^{1}\left(V\right)>0$ for example.

(i) if $J_{1,2}^{0}\left(V\right)>0$ and $J_{1,2}^{1}\left(V\right)>0$, then, the ion channel prefers the cation $S_{1}$ over the cation $S_{2}$, and the small positive permanent charge further enhances this preference;
(ii) if $J_{1,2}^{0}\left(V\right)<0$ and $J_{1,2}^{1}\left(V\right)<0$, then, the ion channel prefers the cation $S_{2}$ over the cation $S_{1}$, and the small positive permanent charge further enhances this preference.

Similar argument can be applied to the case $J_{1,2}^{0}\left(V\right)J_{1,2}^{1}\left(V\right)<0$.

### 3.2. Numerical Simulations

In this part, numerical simulations are performed to provide more intuitive illustrations of some analytical results. To get started, we rewrite the system (8) and (9) as a system of first order ordinary differential equations. Upon introducing $u=\varepsilon \dot{\phi }$, one has

(50) $$\begin{array}{c} \begin{array}{cc} \varepsilon \dot{\phi }= & u,\;\varepsilon \dot{u}=-z_{1}c_{1}-z_{2}c_{2}-z_{3}c_{3}-Q\left(x\right)-\varepsilon \frac{h_{x}\left(x\right)}{h(x)}u,\\ \varepsilon {\dot{c}}_{1}= & -z_{1}c_{1}u-\varepsilon \frac{J_{1}}{h(x)},\;\varepsilon {\dot{c}}_{2}=-z_{2}c_{2}u-\varepsilon \frac{J_{2}}{h(x)},\;\varepsilon {\dot{c}}_{3}=-z_{3}c_{3}u-\varepsilon \frac{J_{3}}{h(x)},\\ {\dot{J}}_{1}= & {\dot{J}}_{2}={\dot{J}}_{3}=0 \end{array} \end{array}$$

with boundary conditions

(51) $$\phi \left(0\right)=V,\;c_{k}\left(0\right)=L_{k};\;\phi \left(1\right)=0,\;c_{k}\left(1\right)=R_{k},\;k=1,2,3.$$

In our simulations to system (50) and (51), we take $z_{1}=z_{2}=-z_{3}=1,\;D_{1}=2,$$D_{2}=8,\;D_{3}=10,\;\varepsilon =0.01,\;Q_{0}=0.01,$

$$Q\left(x\right)=\left\{\begin{array}{cc} 0, & 0<x<a,\\ Q_{0}, & a<x<b,\\ 0, & b<x<1, \end{array}\right.\;\mathrm{and}\;h\left(x\right)=\left\{\begin{array}{cc} \pi {\left(-x+r_{0}+a\right)}^{2}, & 0\leq x<a,\\ \pi r_{0}^{2}, & a\leq x<b,\\ \pi {\left(x+r_{0}-b\right)}^{2}, & b\leq x<1. \end{array}\right.$$

**Remark** **3.** *The choice of h(x) is based on the fact that the ion channel is cylindrical-like, and the variable cross-section area is chosen to reflect the fact that the channel is not uniform and much narrower in the neck (a<x<b) than other regions [9]. We further take $r_{0}=0.5$ and the function h(x) is then continuous at the jumping points x=a and x=b. Different models for h(x) may be chosen, and similar numerical results should be obtained.*

Our numerical simulations consist of two parts focusing on some cases discussed in Section 3.1.1, Section 3.1.2 and Section 3.1.3, respectively, and further verify some analytical results stated in Theorems 3, 4, 6, 7 and 9 for some carefully selected system parameters. Other related results can also be numerically illustrated by choosing different parameter values, and we leave that to interested readers.

Recall that

$$\begin{array}{cc} \; & L=L_{1}+L_{2},\;R=R_{1}+R_{2},\;L_{d}=D_{1}L_{1}-D_{2}L_{2},\;R_{d}=D_{1}R_{1}-D_{2}R_{2},\\ \; & \alpha =\frac{H(a)}{H(1)},\;\beta =\frac{H(b)}{H(1)},\;t=\frac{L}{R}. \end{array}$$

**Figure 2:**
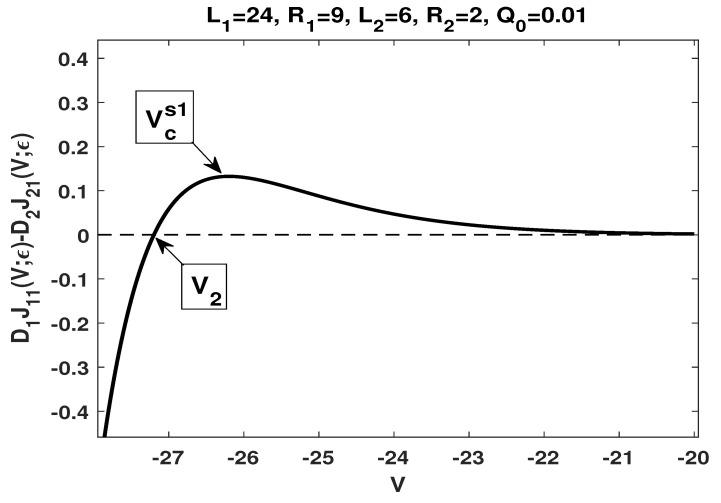
Identification of critical potentials $V_{2}$ and $V_{c}^{s1}$ and approximation of $J_{1,2}^{1}\left(V\right)$ with $\frac{D_{1}}{D_{2}}=\frac{L_{2}}{L_{1}}$ for ε=0.01.

**Figure 3:**
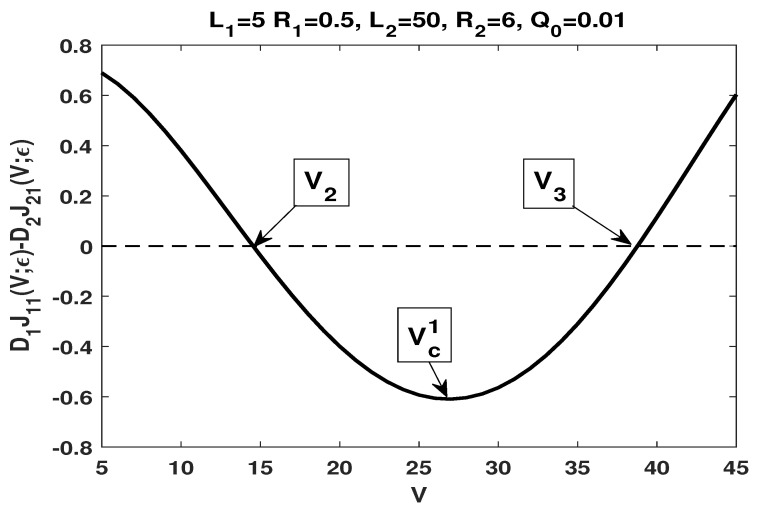
Identification of critical potentials $V_{2},V_{3}$ and $V_{c}^{1}$ and approximation of $J_{1,2}^{1}\left(V\right)$ with $\frac{D_{1}}{D_{2}}<\min\left\{\frac{L_{2}}{L_{1}},\frac{R_{2}}{R_{1}}\right\}$ for ε=0.01.

**Figure 4:**
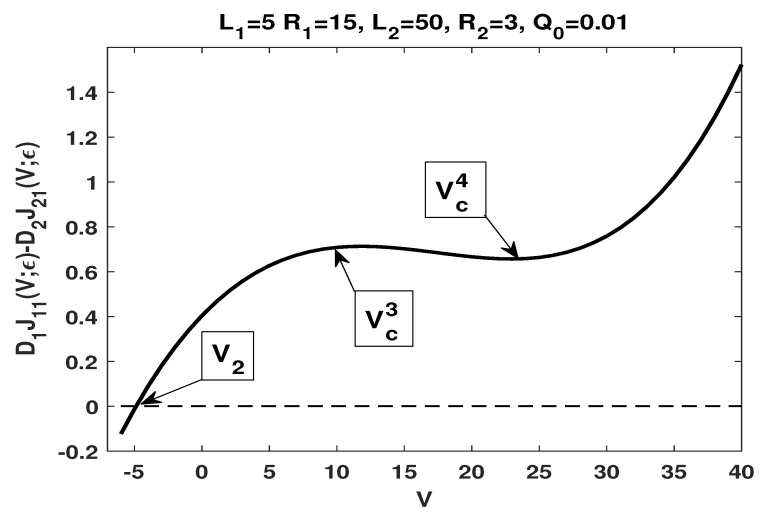
Identification of critical potentials $V_{2},\;V_{c}^{3}$ and $V_{c}^{4}$ and approximation of $J_{1,2}^{1}\left(V\right)$ with $\frac{R_{2}}{R_{1}}<\frac{D_{1}}{D_{2}}<\frac{L_{2}}{L_{1}}$ for ε=0.01.

It turns out that our numerical simulations with nonzero but small ε are consistent with our analytical results. To be specific,

(i) By choosing $L_{1}=24,\;L_{2}=6,\;R_{1}=9,\;R_{2}=2,\;a=1/3$ and b=0.7, one has
  $$\begin{array}{cc} \alpha = & 0.265139,\;\beta =0.751381,\;\frac{D_{1}}{D_{2}}=\frac{1}{4},\;\frac{L_{2}}{L_{1}}=\frac{1}{4},\;t=\frac{30}{11},\;\beta_{1}=0.804956,\\ \gamma (t)= & 0.57531,\;R_{d}=2>0. \end{array}$$
  It follows that
  $$\begin{array}{cc} \; & \frac{D_{1}}{D_{2}}=\frac{L_{2}}{L_{1}},\;\alpha <\gamma \left(t\right)<\alpha -\frac{z}{z_{3}\ln t}=1.261902,\;\beta \in \left(\alpha ,\beta_{1}\right), \end{array}$$
  which is consistent with the conditions stated in (ii) of Lemma 8, and indicates that 1−B<0. Together with Lemma 7, we have A(1−B)>0. Therefore, $A\left(1-B\right)R_{d}>0$, which is consistent with the condition (i) stated in Theorems 3 and 4, respectively.
  Our numerical results show that (see Figure 2) $J_{1,2}^{1}\left(V;\varepsilon \right)$, approximation of $J_{1,2}^{1}\left(V\right)=D_{1}J_{11}\left(V\right)-D_{2}J_{21}\left(V\right)$ defined in (48), which is given by
  $$J_{1,2}^{1}\left(V;\varepsilon \right)=D_{1}\left[J_{1}\left(V;Q_{0};\varepsilon \right)-J_{1}\left(V;0;\varepsilon \right)\right]-D_{2}\left[J_{2}\left(V;Q_{0};\varepsilon \right)-J_{2}\left(V;0;\varepsilon \right)\right],$$
  has a unique zero $V_{2}$, and a critical point $V_{c}^{s1}>V_{2}$ such that $J_{1,2}^{1}\left(V;\varepsilon \right)<0$ if $V<V_{2}$ and $J_{1,2}^{1}\left(V;\varepsilon \right)>0$ if $V>V_{2}$; furthermore, $J_{1,2}^{1}\left(V;\varepsilon \right)$ increases in the potential *V* if $V<V_{c}^{s1}$, and decreases in the potential *V* if $V>V_{c}^{s1}$.
  We remark that the numerical result is consistent with our analytical result, more precisely, statement (i) of both Theorems 3 and 4.
(ii) By choosing $L_{1}=5,\;L_{2}=50,\;R_{1}=0.5,\;R_{2}=6,\;a=1/3$ and b=1/2, one has
  $$\begin{array}{cc} \alpha = & 0.324324,\;\beta =0.594595,\;\frac{D_{1}}{D_{2}}=\frac{1}{4},\;\frac{L_{2}}{L_{1}}=10,\;\frac{R_{2}}{R_{1}}=12,\;t=8.461538,\\ \frac{L_{d}}{R_{d}}= & 8.297872,\;\beta_{1}=0.826171,\;\gamma \left(t\right)=0.665753. \end{array}$$
  It follows that
  $$\begin{array}{cc} \; & \frac{D_{1}}{D_{2}}<\min\left\{\frac{L_{2}}{L_{1}},\;\frac{R_{2}}{R_{1}}\right\},\;\alpha <\gamma \left(t\right)<\alpha -\frac{z}{z_{3}\ln t}=0.792592,\\ \; & \beta \in \left(\alpha ,\beta_{1}\right),\;t>\frac{L_{d}}{R_{d}}>1, \end{array}$$
  which is consistent with the conditions stated in (ii) of Corollary 4.
  Our numerical results show that (see Figure 3) $J_{1,2}^{1}\left(V;\varepsilon \right)$, approximation of $J_{1,2}^{1}\left(V\right)=D_{1}J_{11}\left(V\right)-D_{2}J_{21}\left(V\right)$ defined in (48), which is given by
  $$J_{1,2}^{1}\left(V;\varepsilon \right)=D_{1}\left[J_{1}\left(V;Q_{0};\varepsilon \right)-J_{1}\left(V;0;\varepsilon \right)\right]-D_{2}\left[J_{2}\left(V;Q_{0};\varepsilon \right)-J_{2}\left(V;0;\varepsilon \right)\right],$$
  has two zeros $V_{2}$ and $V_{3}$ with $V_{2}<V_{3}$, and a critical point $V_{c}^{1}$ between $V_{2}$ and $V_{3}$ such that $J_{1,2}^{1}\left(V;\varepsilon \right)>0$ if $V<V_{2}$ or $V>V_{3}$, and $J_{1,2}^{1}\left(V;\varepsilon \right)<0$ if $V_{2}<V<V_{3}$; furthermore, $J_{1,2}^{1}\left(V;\varepsilon \right)$ decreases in the potential *V* if $V<V_{c}^{1}$, and increases in the potential *V* if $V>V_{c}^{1}$.
  We remark that the numerical result is consistent with our analytical result, more precisely, statement (iii) of Theorem 6 and statement (i) of Theorem 7.
(iii) By choosing $L_{1}=5,\;L_{2}=50,\;R_{1}=15,\;R_{2}=3,\;a=1/3$ and b=1/2, one has
  $$\begin{array}{cc} \alpha = & 0.324324,\;\beta =0.594595,\;\frac{D_{1}}{D_{2}}=\frac{1}{4},\;\frac{L_{2}}{L_{1}}=10,\;\frac{R_{2}}{R_{1}}=\frac{1}{5},\;t=\frac{55}{18},\\ \beta_{1}= & 0.782204,\;\gamma (t)=0.591200. \end{array}$$
  It follows that
  $$\begin{array}{c} \frac{R_{2}}{R_{1}}<\frac{D_{1}}{D_{2}}<\frac{L_{2}}{L_{1}},\;\alpha <\gamma \left(t\right)<\alpha -\frac{z}{z_{3}\ln t}=1.219610,\;\beta \in \left(\alpha ,\beta_{1}\right). \end{array}$$
  From Lemma 7, one has A<0 and from Lemma 8 (statement (ii1)), one has 1−B<0, and hence, A(1−B)>0, which satisfies the condition required in the statement (i) of Theorem 9.
  Our numerical results show that (see Figure 4) $J_{1,2}^{1}\left(V;\varepsilon \right)$ has one zero $V_{2}$ and two critical points $V_{c}^{3}$ and $V_{c}^{4}$ with $V_{c}^{3}<V_{c}^{4}$ such that $J_{1,2}^{1}\left(V;\varepsilon \right)>0$ if $V>V_{2}$ and $J_{1,2}^{1}\left(V;\varepsilon \right)<0$ if $V<V_{2}$; furthermore, $J_{1,2}^{1}\left(V;\varepsilon \right)$ increases in the potential *V* if $V<V_{c}^{3}$, decreases in the potential *V* if $V_{c}^{3}<V<V_{c}^{4}$, and increases in the potential *V* if $V>V_{c}^{4}$.
  We remark that the numerical result is consistent with our analytical result stated in (i) of Theorem 9.

## 4. Concluding Remarks

We study a one-dimensional steady-state Poisson–Nernst–Planck system with three ion species, two cations having the same valence and one anion. Nonzero but small permanent charge, the major structural quantity of an ion channel, is included in the model. The cross-section area of the channel is included in the system, and it provides certain information of the geometry of the three-dimensional channel, which plays crucial roles in our analysis. Two specific structures of the PNP model

(i) the existence of a complete set of first integrals (Proposition 1, first statement (i) in Proposition 4 and Proposition 5, respectively);
(ii) the observation made in Section 2.1.2 allows one to transform the limit slow system (32) to a linear system (33) with constant coefficients;

allows one to reduce the singularly perturbed boundary value problem to an algebraic system-the governing system (40). The significance of the governing system is:

(i) it includes almost all relevant physical parameters;
(ii) once a solution of the governing system is obtained, the singular orbit (the zeroth order approximation (in ε) solution of the boundary value problem) can be readily determined.

Based on these specific structures of this concrete model, under the framework of the geometric singular perturbation theory, a singular orbit is obtained, from which explicit expressions of $J_{k0}$ and $J_{k1}$ are extracted. This makes it possible for one to further analyze the competition between cations, which further depend on the complicated nonlinear interplays among system parameters, such as the diffusion coefficients $\left(D_{1},D_{2}\right)$, the channel geometry in terms of (α,β), the boundary conditions $(L_{k},R_{k};V),\;k=1,2,3$, particularly, the interaction among $\frac{D_{1}}{D_{2}},\;\frac{R_{2}}{R_{1}}$ and $\frac{L_{2}}{L_{1}}$ plays a critical role in characterizing the competition between cations (Section 3.1.1, Section 3.1.2 and Section 3.1.3). Among others, we find

(i) As functions of the membrane potential *V* (fixing other system parameters),
  (i1) $J_{1,2}^{1}=D_{1}J_{11}-D_{2}J_{21}$ and $\partial_{V}J_{1,2}^{1}$ can be positive (resp. negative), which further depends on the nonlinear interaction among boundary concentrations and diffusion coefficients. The sign of $J_{1,2}^{1}$ provides critical information related to the selectivity phenomena of ion channels, while the sign of $\partial_{V}J_{1,2}$ provides efficient ways to further adjust/control the preference of the ion channel over distinct cation (Characterized in Theorems 2–9);
  (i2) $|J_{1,2}|$, the magnitude of $J_{1,2}$, which is equivalent to $J_{1,2}^{0}J_{1,2}^{1}$ for small positive $Q_{0}$, is analyzed, which provides further information of the ion channel over distinct cations (Theorems 10 and 11).
(ii) Critical potentials that balance the small permanent charge effects on $J_{1,2}^{1}$ (such as $V_{2}$ and $V_{3}$) are identified (Definition 1). Those critical potentials can be experimentally estimated. Taking $V_{2}$ for example, one can take an experimental curve as $J_{1,2}\left(V;Q_{0}\right)$ and numerically (or analytically) compute $J_{1,2}^{0}\left(V;0\right)$ for ideal case that allows one to get an estimate of $V_{2}$ by considering the zeros of $J_{1,2}\left(V;Q_{0}\right)-J_{1,2}^{0}\left(V;0\right)$. The critical potentials play critical roles in characterizing permanent charge effects on ionic flows through membrane channels.

Finally, we comment that the setup in this work is relatively simple, and may raise the concern about the feasibility. Indeed, cPNP is known to be reliable when the ionic mixture is dilute, but with more ion species and nonzero permanent charges included, the ionic mixture would be crowded. On the other hand, the setup is reasonable for semi-conductor problems and for synthetic channels. The detailed analysis in this work is not only important in understanding the behavior of such fluxes across channels but can also better help in understanding the fundamental mechanistic principles of metabolite fluxes in engineered synthetic biological channels [52]. Furthermore, the study in this work is the first step for analysis of more realistic models. The simple model considered in this work allows us to obtain more explicit expressions of the ionic fluxes in terms of physical parameters of the problem so that we are able to extract concrete information on small permanent charge effects. Moreover, the analysis in this simpler setting provides important insights for the analysis and numerical studies of more realistic models.

## References

[1] Eisenberg B. (2012). Ions in Fluctuating Channels: Transistors Alive. Fluct. Noise Lett..

[2] Eisenberg B., Rice S.A. (2011). Crowded charges in ion channels. Advances in Chemical Physics.

[3] Gillespie D. (1999). A Singular Perturbation Analysis of the Poisson–Nernst–Planck System: Applications to Ionic Channels. Ph.D. Thesis.

[4] Mofidi H., Eisenberg B., Liu W. (2020). Effects of Diffusion Coefficients and Permanent Charge on Reversal Potentials in Ionic Channels. Entropy.

[5] Bates P.W., Chen J., Zhang M. (2020). Dynamics of ionic flows via Poisson–Nernst–Planck systems with local hard-sphere potentials: Competition between cations. Math. Biosci. Eng..

[6] Bates P.W., Jia Y., Lin G., Lu H., Zhang M. (2017). Individual flux study via steady-state Poisson–Nernst–Planck systems: Effects from boundary conditions. SIAM J. Appl. Dyn. Syst..

[7] Eisenberg B., Liu W. (2007). Poisson–Nernst–Planck systems for ion channels with permanent charges. SIAM J. Math. Anal..

[8] Ji S., Eisenberg B., Liu W. (2019). Flux ratios and channel structures. J. Dyn. Differ. Equ..

[9] Ji S., Liu W., Zhang M. (2015). Effects of (small) permanent charges and channel geometry on ionic flows via classical Poisson–Nernst–Planck models. SIAM J. Appl. Math..

[10] Liu W. (2005). Geometric singular perturbation approach to steady-state Poisson–Nernst–Planck systems. SIAM J. Appl. Math..

[11] Liu W. (2009). One-dimensional steady-state Poisson–Nernst–Planck systems for ion channels with multiple ion species. J. Differ. Equ..

[12] Liu W., Xu H. (2015). A complete analysis of a classical Poisson–Nernst–Planck model for ionic flow. J. Differ. Equ..

[13] Park J.-K., Jerome J.W. (1997). Qualitative properties of steady-state Poisson–Nernst–Planck systems: Mathematical study. SIAM J. Appl. Math..

[14] Wen Z., Zhang L., Zhang M. (2020). Dynamics of classical Poisson–Nernst–Planck systems with multiple cations and boundary layers. J. Dyn. Differ. Equ..

[15] Eisenberg B. (2003). Ion Channels as Devices. J. Comput. Electr..

[16] Eisenberg B. (2003). Proteins, channels, and crowded ions. Biophys. Chem..

[17] Im W., Roux B. (2002). Ion permeation and selectivity of OmpF porin: A theoretical study based on molecular dynamics, Brownian dynamics, and continuum electrodiffusion theory. J. Mol. Biol..

[18] Roux B., Allen T.W., Berneche S., Im W. (2004). Theoretical and computational models of biological ion channels. Q. Rev. Biophys..

[19] Schuss Z., Nadler B., Eisenberg R.S. (2001). Derivation of Poisson and Nernst-Planck equations in a bath and channel from a molecular model. Phys. Rev. E.

[20] Barcilon V. (1992). Ion flow through narrow membrane channels: Part I. SIAM J. Appl. Math..

[21] Hyon Y., Eisenberg B., Liu C. (2010). A mathematical model for the hard sphere repulsion in ionic solutions. Commun. Math. Sci..

[22] Hyon Y., Fonseca J., Eisenberg B., Liu C. (2011). A new Poisson–Nernst–Planck Equation (PNP-FS-IF) for charge inversion near walls. Biophys. J..

[23] Hyon Y., Liu C., Eisenberg B. (2012). PNP equations with steric effects: A model of ion flow through channels. J. Phys. Chem. B.

[24] Biesheuvel P.M. (2011). Two-fluid model for the simultaneous flow of colloids and fluids in porous media. J. Colloid Interface Sci..

[25] Chen D., Eisenberg R., Jerome J., Shu C. (1995). Hydrodynamic model of temperature change in open ionic channels. Biophys. J..

[26] Eisenberg B., Hyon Y., Liu C. (2010). Energy variational analysis of ions in water and channels: Field theory for primitive models of complex ionic fluids. J. Chem. Phys..

[27] Fair J.C., Osterle J.F. (1971). Reverse Electrodialysis in charged capillary membranes. J. Chem. Phys..

[28] Gross R.J., Osterle J.F. (1968). Membrane transport characteristics of ultra fine capillary. J. Chem. Phys..

[29] Sasidhar V., Ruckenstein E. (1981). Electrolyte osmosis through capillaries. J. Colloid Interface Sci..

[30] Wei G.W. (2010). Differential geometry based multiscale models. Bull. Math. Biol..

[31] Wei G.W., Zheng Q., Chen Z., Xia K. (2012). Variational multiscale models for charge transport. SIAM Rev..

[32] Barcilon V., Chen D.-P., Eisenberg R.S. (1992). Ion flow through narrow membrane channels: Part II. SIAM J. Appl. Math..

[33] Abaid N., Eisenberg R.S., Liu W. (2008). Asymptotic expansions of I-V relations via a Poisson–Nernst–Planck system. SIAM J. Appl. Dyn. Syst..

[34] Barcilon V., Chen D.-P., Eisenberg R.S., Jerome J.W. (1997). Qualitative properties of steady-state Poisson–Nernst–Planck systems: Perturbation and simulation study. SIAM J. Appl. Math..

[35] Lee C.-C., Lee H., Hyon Y., Lin T.-C., Liu C. (2011). New Poisson-Boltzmann type equations: One-dimensional solutions. Nonlinearity.

[36] Liu W., Wang B. (2010). Poisson–Nernst–Planck systems for narrow tubular-like membrane channels. J. Dyn. Differ. Equ..

[37] Singer A., Gillespie D., Norbury J., Eisenberg R.S. (2008). Singular perturbation analysis of the steady-state Poisson–Nernst–Planck system: Applications to ion channels. Eur. J. Appl. Math..

[38] Singer A., Norbury J. (2009). A Poisson–Nernst–Planck model for biological ion channels–an asymptotic analysis in a three-dimensional narrow funnel. SIAM J. Appl. Math..

[39] Wang X.-S., He D., Wylie J., Huang H. (2014). Singular perturbation solutions of steady-state Poisson–Nernst–Planck systems. Phys. Rev. E.

[40] Nooner W., Eisenberg R.S. (1998). Ion permeation and glutamate residues linked by Poisson–Nernst–Planck theory in L-type Calcium channels. Biophys. J..

[41] Gillespie D., Nonner W., Eisenberg R.S. (2002). Coupling Poisson–Nernst–Planck and density functional theory to calculate ion flux. J. Phys. Condens. Matter.

[42] Kilic M.S., Bazant M.Z., Ajdari A. (2007). Steric effects in the dynamics of electrolytes at large applied voltages. II. Modified Poisson–Nernst–Planck equations. Phys. Rev. E.

[43] Lin G., Liu W., Yi Y., Zhang M. (2013). Poisson–Nernst–Planck systems for ion flow with density functional theory for local hard-sphere potential. SIAM J. Appl. Dyn. Syst..

[44] Liu W., Tu X., Zhang M. (2012). Poisson–Nernst–Planck systems for ion flow with density functional theory for hard-sphere potential: I-V relations and critical potentials. Part II: Numerics. J. Dyn. Differ. Equ..

[45] Rouston D.J. (1990). Bipolar Semiconductor Devices.

[46] Warner R.M. (2001). Microelectronics: Its unusual origin and personality. IEEE Trans. Electron. Devices.

[47] Eisenberg B., Liu W. (2017). Relative dielectric constants and selectivity ratios in open ionic channels. Mol. Based Math. Biol..

[48] Jones C. (1995). Geometric singular perturbation theory. Dynamical Systems (Montecatini Terme, 1994).

[49] Fenichel N. (1979). Geometric singular perturbation theory for ordinary differential equations. J. Differ. Equ..

[50] Tin S.-K., Kopell N., Jones C. (1994). Invariant manifolds and singularly perturbed boundary value problems. SIAM J. Numer. Anal..

[51] Jones C., Kopell N. (1994). Tracking invariant manifolds with differential forms in singularly perturbed systems. J. Differ. Equ..

[52] Mohammad M.M., Prakash S., Matouschek A., Movileanu L. (2008). Controlling a single protein in a nanopore through electrostatic teaps. J. Am. Chem. Soc..

